# CCL8 as a promising prognostic factor in diffuse large B-cell lymphoma *via* M2 macrophage interactions: A bioinformatic analysis of the tumor microenvironment

**DOI:** 10.3389/fimmu.2022.950213

**Published:** 2022-08-22

**Authors:** Xiaoli Lou, Ke Zhao, Jingze Xu, Lixiong Shuai, Hui Niu, Zhifei Cao, Juan Wang, Yongsheng Zhang

**Affiliations:** ^1^ Department of Pathology, The Second Affiliated Hospital of Soochow University, Suzhou, China; ^2^ Department of Pathology, The Affiliated Jiangyin Hospital of Nantong Universtiy, Jiangyin, China; ^3^ Department of Pathology, Suzhou Wuzhong People’s Hospital, Suzhou, China

**Keywords:** Diffuse large B-cell lymphoma, tumor microenvironment, estimate, CIBERSORT, macrophage, C-C motif chemokine ligand 8

## Abstract

**Backgrounds:**

Prior investigations of the tumor microenvironment (TME) of diffuse large B-cell lymphoma (DLBCL) have shown that immune and stromal cells are key contributing factors to patients’ outcome. However, challenges remain in finding reliable prognostic biomarkers based on cell infiltration. In this study, we attempted to shed some light on chemokine C–C motif chemokine ligand 8 (CCL8) in DLBCL *via* interaction with M2 macrophages.

**Methods:**

The Estimation of STromal and Immune cells in MAlignant Tumor tissues using Expression data (ESTIMATE) algorithm was applied to evaluate immune and stromal scores from transcriptomic profiles of 443 DLBCL samples from The Cancer Genome Atlas (TCGA) and GSE10846 datasets. Immune cell infiltration (ICI) clusters were obtained based on different immune cell infiltrations of each sample, and gene clusters were derived through differentially expressed genes (DEGs) between the distinct ICI clusters. Five immune-related hub genes related to overall survival (OS) and clinical stages were obtained by COX regression analysis and protein–protein interaction (PPI) network construction then verified by quantitative real-time PCR (qPCR) and immunofluorescence staining in the FFPE tissues. The Gene Ontology (GO), Kyoto Encyclopedia of Genes and Genomes (KEGG), and TIMER websites were employed to explore the biological functions of CCL8-related DEGs. Uni- and multivariable Cox regression analyses were performed to analyze CCL8 as an independent prognostic risk factor in GSE10846 and were verified in other independent GEO cohorts.

**Results:**

A higher stromal score was associated with favorable prognosis in DLBCL. Patients in the ICI B cluster and gene B clusters had a better follow-up status with a higher programmed death ligand 1 (PD-L1) and cytotoxic T-lymphocyte antigen 4 (CTLA4) expression. Most of ICI-related DEGs were enriched for immune-related signaling pathways. Five hub genes with a distinct prognosis association were identified, including CD163, which is a biomarker of M2 macrophages, and CCL8. Abundant M2 macrophages were discovered in the high-CCL8 expression group. The functional analysis indicated that CCL8 is a key component of immune-related processes and secretory granule groups. Cox regression analysis and data from other GSE datasets yielded additional evidence of the prognostic value of CCL8 in DLBCL.

**Conclusions:**

CCL8 has been implicated in macrophage recruitment in several solid tumors, and only a few reports have been published on the role of CCL8 in the pathogenesis of hematological malignancies. This article attempted to find out TME-related genes that associated with the survival in DLBCL patients. CCL8 was identified to be involved in immune activities. Importantly, a series of bioinformatics analysis indicated that CCL8 might become an effective target for DLBCL, which interacts with M2 macrophage and immune checkpoint. The potential related mechanisms need to be further elucidated.

## Introduction

DLBCL is the most prevalent form of aggressive non-Hodgkin lymphomas (NHLs) and represents a group of heterogenous diseases with varying outcomes that have different properties including clinical features, immune cell infiltration (ICI), and immunity-related gene expression (1). One-third of patients with DLBCL will relapse despite the conventional chemotherapy (2). The mechanistic basis of immune escape in DLBCL is poorly understood and lacks a therapeutic target.

The tumor microenvironment (TME) has become one of the most emerging prospective fields in cancer research (3). Considerable data *via* gene sequencing and expression profiling technology indicate that the TME, consisting of tumor-related stromal cells, infiltrating immune cells, and other normal epithelial cells, is a key aspect in the prognosis of lymphoma, including DLBCL (4–7). A wide range of studies have shown the role of stromal cells and immune cells on clinical features in solid tumors (8–10). Among them, macrophages are the most plastic type of immune cells and some epigenetic modifications on macrophages accelerate the tumor process (7). Further, cancer cells can functionally modify their TME by secreting various chemokines. In central nervous system lymphoma, gliosis retains tumor cells through secreting CCL19 (11). Inhibitors targeting immune checkpoints such as PD-L1 and CTLA4 represent a promising approach for DLBCL (12, 13). Given the importance of the TME, it is of great interest to construct gene regulatory networks based on immune and stromal components for exploring the biological processes of DLBCL development.

**Figure 1:**
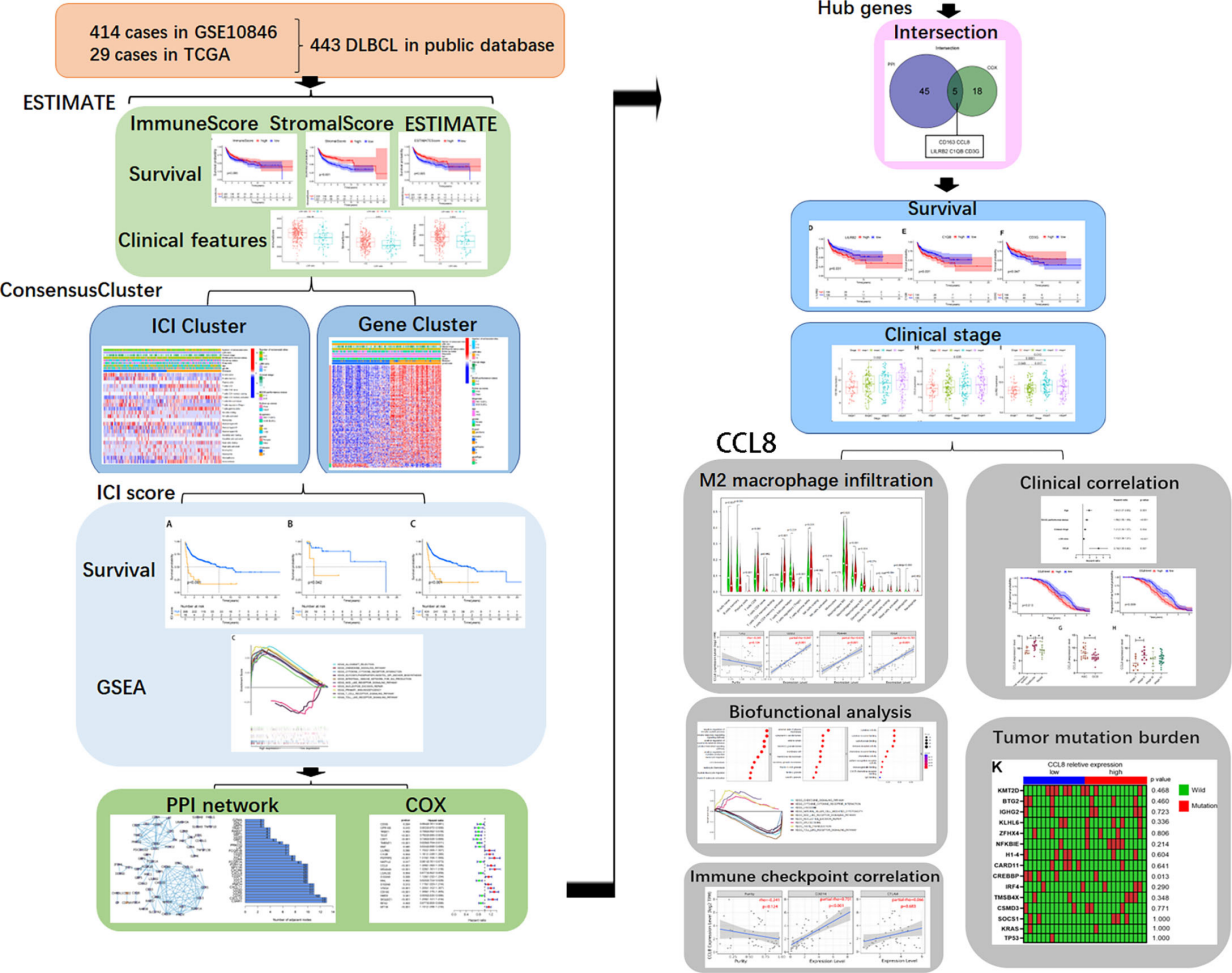
Workflow of the study.

With growing availability of multilevel expression data from tumor tissues, extracting and integrating the large datasets, such as TCGA and Gene Expression Omnibus (GEO), is a new opportunity for providing a more comprehensive understanding of cancers (14). In this study, we used ESTIMATE algorithms to estimate the immune and stromal scores of a series of DLBCL tissues from TCGA and GEO databases and analyzed a stromal-immune score-based hub gene, CCL8, for prognosis stratification in DLBCL with clinical specimen validation (**Figure 1**). Using this method, we can predict the effects on prognosis and the benefit of immunotherapy in DLBCL patients based on the immune signature.

## Methods

### Data collection and preparation

We obtained transcriptome profiling and clinical data of a total of 449 DLBCL cases and from TCGA Data Portal (n = 29) (https://portal.gdc.cancer.gov/) and GEO datasets (GSE10846, n = 420) (https://www.ncbi.nlm.nih.gov/gds/) (**Table 1**). The transcriptional values in fragments per kilobase of exon per million reads mapped (FPKM) of TCGA data were Log2-transformed into transcripts per kilobase of exon model per million mapped reads (TPM) *via* the R package “limma” in R 3.6.0. For the ICI cluster and ICI-related gene cluster, we used data from 283 cases from the GSE10846 and TCGA datasets and excluded 166 cases with incomplete clinical information. We used the data of 312 samples for ICI-related DEG analysis and removed 108 samples with missing DEG expression data.

**Table 1:** The survey of database.

Database	Platform	Tissue (Human sapiens)	Samples (number)	Experiment type	Author
GSE10846	GPL570	lymph node	420	Array	Louis M. Staudt
GSE136971	GPL570	lymph node	221	Array	Sylvain Mareschal
GSE10524	GPL570	lymph node	40	Array	Marije Booman
GSE64555	GPL570	lymph node	40	Array	Kim Linton
GSE114175	GPL24975	lymph node	52	Array	Philipp Kämmer
TCGA		lymph node	29	Array	

For the analysis of the clinical biopsies, eight DLBCL samples and four normal lymph node samples were collected from the Second Affiliated Hospital of Soochow University. All the patients did not receive radiotherapy or chemotherapy before surgery. Tissue samples were stored in 10% formalin solution for paraffin embedding. All samples were diagnosed as DLBCL or normal lymph node by two pathologists following the World Health Organization (WHO) guidelines for diagnosis. The project was approved by the ethics committee of the Second Affiliated Hospital of Soochow University (Ethics ID: JD-HG-2022-37).

### ESTIMATE algorithm and identification of stromal and immune groups

The stromal and immune scores were derived by standardizing the expression matrix using ESTIMATE algorithms based on the expression of two independent sets including 141 genes that represent the degree of tumor stromal and immune cell infiltrations (15). The analysis methods were consolidated *via* the “estimate” R package in R 3.6.0. The median was used as the cutoff point for the high and low immune, stromal, and ESTIMATE score groups. Comparisons of the immune, stromal, and ESTIMATE scores between each characteristic, such as age, gender, ECOG, LDH ratio, number of extranodal sites, and stage, were conducted using unpaired t-test.

### ICI and gene cluster analysis

The “ConsensusClusterPlus” R package in R 3.6.0 was performed to identify the optimal clustering number (κ) chosen from 2 to 9 depending on the consensus matrix, consensus cumulative distribution function (CDF), delta area, and tracking plots. The clustering consensus and item consensus were calculated separately. Finally, heatmaps of associations between genes and clinical characteristics in different clusters were generated using the “pheatmap” R package in R 3.6.0.

### Gene Ontology and KEGG pathway enrichment analyses

Gene Ontology (GO) and KEGG pathway enrichment analyses were performed for the featured genes obtained by the PCA algorithm using “clusterProfiler”, “org.Hs.eg.db”, “enrichplot”, and “ggplot2” packages in R. Terms were considered significantly enriched only with both *P <* 0.05 and q < 1.

### Gene set enrichment analysis

Gene set enrichment analysis (GSEA) (version 4.2.1) was used to identify potential different signaling pathways between two ICI groups. The number of permutations was 100, and a false discovery rate (FDR) <0.05 or normal *P <*0.05 was followed.

### DEGs and ICI score

The “Limma” R package in R 3.6.0 was employed for screening DEGs among ICI subgroups with log fold change (FC) >1 and adjusted P value <0.05. Those DEGs demonstrating positive and negative correlations with gene clusters were named as ICI gene signatures A and B, respectively. The samples were scored for immunity according to the expression of feature genes of each immune cell then divided into two groups by median scores. Principal component 1 (PC1) was considered as the signature score by applying principal component analysis (PCA) after reduced noise or redundant genes in the ICI gene signature *via* the Boruta algorithm. The ICI score of each subject was calculated as follows: ICI score = Ʃ PC1A – PC1B.

### COX regression analysis

Uni- and multivariate Cox proportional hazard models were employed to calculate the hazard ratios (HRs) with a 95% confidence interval (95% CI), which measure the strength of the correlation between dependent variables and overall survival. “Limma” and “survival” R packages in R 3.6.0 were employed for COX regression. P < 0.05 was considered statistically significant.

### PPI network construction of DEGs

A PPI network of DEGs screened from the ICI A group and B group was constructed using the STRING website (https://cn.string-db.org/). The reconstruction was then performed with Cytoscape version 3.6.1, and nodes selected to build the PPI network had confidence levels greater than 0.95.

### Survival analysis

A total of 443 samples with survival time from 0 to 21.78 years were subjected to survival analysis using “survival” and “survminer” in R packages. The Kaplan–Meier method was utilized for plotting survival curves. The corresponding significance of survival curves was quantified by log-rank tests.

### Real-time PCR assay

Total RNA was extracted from cells using 1 ml TRIzol. To isolate mRNA from human tissues, 10-μm paraffin-embedded tissues were deparaffinized by using xylene and followed by the isolation of mRNA using the High Pure FFPET RNA Kit (Magen, Guangzhou, China). qPCR was carried out using the Absolute qPCR SYBR Green Master Mix (Vazyme, Shanghai, China) with the following cycling conditions: 95°C for 10 min, 40 cycles of 95°C for 15 s, and 60°C for 60 s. A StepOnePlus device (Thermo Fisher Scientific) was employed for the detection. Glyceraldehyde-3-phosphate dehydrogenase (GAPDH) was used for normalization. Sequences of all primers are shown in **Supplemental Table 1**.

### Tissue immunofluorescent staining assay

Immunofluorescent staining was performed in paraffin-embedded tissues from lymphoma patients. Tissue sections were deparaffinized, blocked with normal goat serum, and incubated with primary antibodies against CCL-8 (Abcam, MA, USA, Catalog number: ab 155967) or CD163 (Proteintech, Shanghai, China, Catalog number: 16646-1-AP), followed by the addition of anti-rabbit secondary antibodies (Sanying Biotech, Wuhan, China, Catalog number: SA00013-2), counterstained with 4′,6-diamidino-2-phenylindole (DAPI) solution (Roche, Switzerland, Catalog number: 10236276001) and subjected to imaging using a microscope.

### Availability and implementation

The source code, documentation, help, and use cases are available on the GitHub page at https://github.com/sherrylou92/CCL8-DLBCL. It is free for use under the GPL 3 license.

## Results

### Association between estimated infiltrated cells, DLBCL prognosis, and clinical features

**Figure 2:**
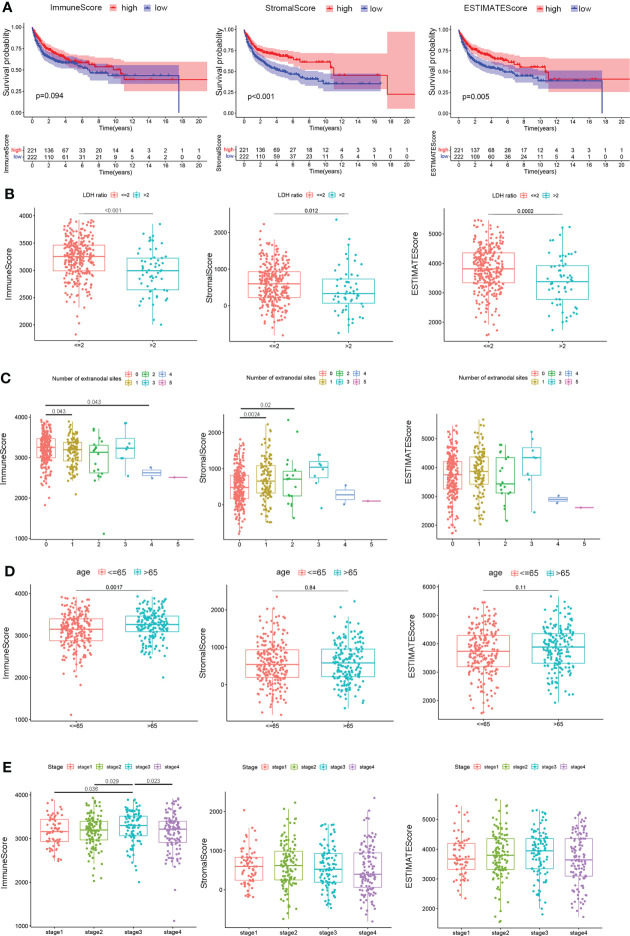
Correlation of scores with the survival and clinical characteristics of patients with DLBCL. **(A)** Kaplan–Meier overall survival curves for 443 TCGA and GSE10846 samples with different immune scores, stromal scores, and ESTIMATE scores. Distribution of immune scores, stromal scores, and ESTIMATE scores in the GEO10846 datasets stratified by **(B)** LDH ratio, **(C)** number of extranodal sites, **(D)** age, and **(E)** clinical stages. Statistical tests were ANOVA tests.

All 449 samples from TCGA and GEO datasets were given stromal and immune scores using the ESTIMATE algorithm, of which survival data were used for 443 cases. These samples were separated into two groups, stratified by the median score. Kaplan–Meier plots indicated that patients with higher stromal score and ESTIMATE score groups showed significantly higher survival probability (*P <* 0.01 and *P =* 0.005, **Figure 2A**). Although the immune score has a limited correlation with patients’ survival (*P =* 0.094, **Figure 2A**), higher immune scores were positively correlated with overall patient survivals within the first decade.

The relationship between immune, stromal, and ESTIMATE scores and pathological characteristics in 412 cases of the GSE10846 dataset, after removing eight cases with no information on clinical characteristics, was then systematically analyzed using the Eastern Cooperative Oncology Group (ECOG), LDH ratio, number of extranodal sites, age, gender, and stage, while indeterminate data were omitted. As depicted in **Figure 2B**, LDH ratios were elevated with lower immune scores (*P <* 0.001), stromal scores (*P =* 0.012), and ESTIMATE scores (*P <* 0.001). In terms of lymph node metastasis, patients with one or two extranodal involvements had higher stromal scores in contrast to no extranodal site invasion (*P =* 0.0024 and *P =* 0.02), while the immune scores were lower for those with four lymph node invasions compared to no other lymph node involvement (P = 0.043, **Figure 2C**). In addition, younger patients (≤65 years) showed decreased immune scores (*P =* 0.0017) but similar stromal scores (*P =* 0.84) and ESTIMATE scores (*P =* 0.11) compared to older patients (> 65 years) (**Figure 2D**). Notably, only immune scores were higher in clinical stage 3 patients compared to stage 1 (*P =* 0.036) or stage 2 (*P =* 0.029), but the scores decreased as the patients progressed to stage 4 (*P =* 0.023, **Figure 2E**). These results provide preliminary evidence that immune components in DLBCL could be related to early survival and clinical characteristics of the patients.

### Characterization of the ICI subgroup and ICI-related genes with pathological characteristics and clinical outcomes in DLBCL

**Figure 3:**
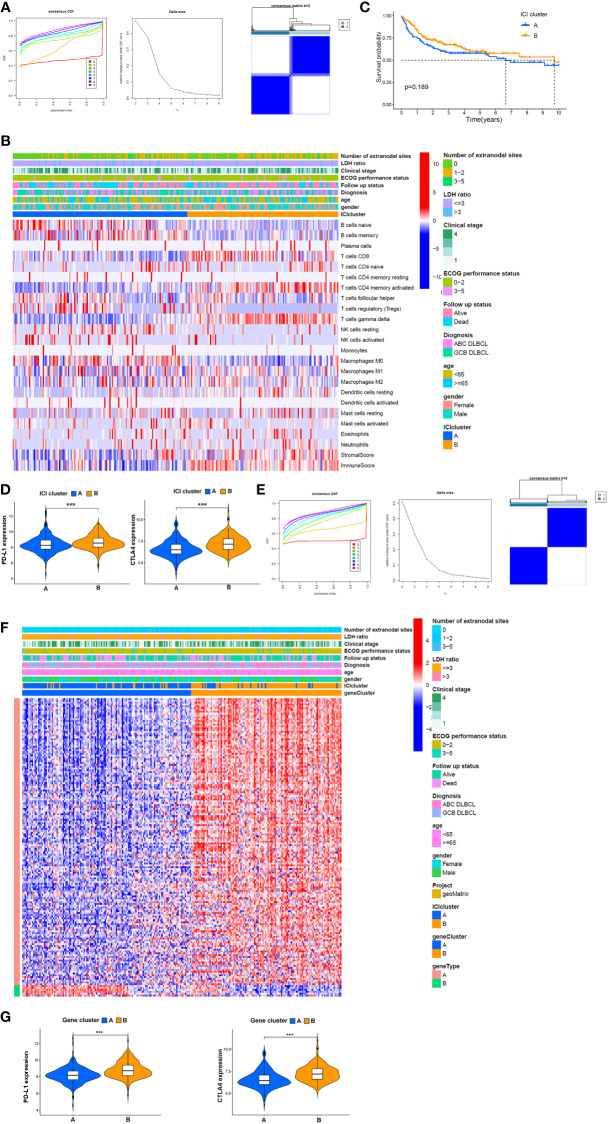
Potential association between TME components and clinical features. **(A)** Left: cumulative distribution function (CDF) of consensus clustering for k = ~2-9; middle: relative change in area under the CDF curve for k = ~2–9; right: relevance between the ICI A and ICI B clusters. **(B)** Heatmap and clinical features of two ICI subgroups defined by 22 immune cell proportions. **(C)** OS analysis of different ICI subgroups *via* a Kaplan–Meier plot. **(D)** Expression of PD-L1 and CTLA4 in patients from distinct ICI subgroups. **(E)** Left: k = 2–9 for the CDF of the consensus clusters. Middle: the area variation under the CDF curve from k = 2–9. Right: association between gene clusters A and B, **(F)** Heatmap of association between the expression of DEGs in gene clusters, ICI subgroups, and clinical features. **(G)** Comparison of the expression of PD-L1 and CTLA4 between patients in different gene clusters. ***P<0.001.

To investigate the association between immune cell infiltration and clinical features, 254 samples from the GSE10846 dataset and 29 samples from TCGA dataset, excluding 166 cases with incomplete clinical information, were separated into different ICI categories, and the value of k = 2 was chosen based on similarities in the expression of immune cells (**Figure 3A**). **Figure 3B** shows a heatmap of clinical characteristic comparison between the two subgroups. Patients in the ICI B groups had better follow-up status and less extranodal site involvement. The 22 kinds of TICs in each sample are also presented. Among them, naïve B cells, memory B cells, Treg cells, resting NK cells, and M0 macrophages were at high levels in the ICI A group, while CD8+ T cells, active memory CD4+ T cells, and γδ T cells were less abundant. No significant correlation with survival probability was obtained between ICI A and B groups at 3, 5, 10, or 20 years (**Supplemental Figures 1A–D**); however, the ICI B group had a longer median survival time and higher survival rate than the ICI A group (ICI A group *P =* 7.49 years, ICI B group *P =* 17.60 years; **Figure 3C**).

PD-L1 and CTLA4 are immune checkpoints, and their inhibitors are often used as a combination therapy in DLBCL treatment. Here, we found that PD-L1 and CTLA4 were more highly expressed in the ICI B group (**Figure 3D**). To further investigate the clinical implications of these ICI subgroups, ICI-related DEGs were filtered by logFC > 2 and *P <* 0.05. One hundred fifty-seven genes positively correlated with ICI; the related gene subgroups were called ICI gene signature A. Seven genes with negative correlations were named ICI gene signature B (**Supplemental Table 2**). ICI-related genes were clustered depending on the DEG expression in each sample. We chose 2 as the value of k, and all samples were divided into two clusters (**Figure 3E**). The heatmap showed that gene cluster A mainly exhibited a higher ECOG performance status at the time of diagnosis showing that patients in this cluster were more likely to succumb to the disease compared with gene cluster B (**Figure 3F**). Also, PD-L1 (*P <* 0.001) and CTLA4 (*P <* 0.001) were all expressed in lower amounts in gene cluster A compared to cluster B (**Figure 3G**).

### Functional correlation analysis of ICI-related DEGs in DLBCL

**Figure 4:**
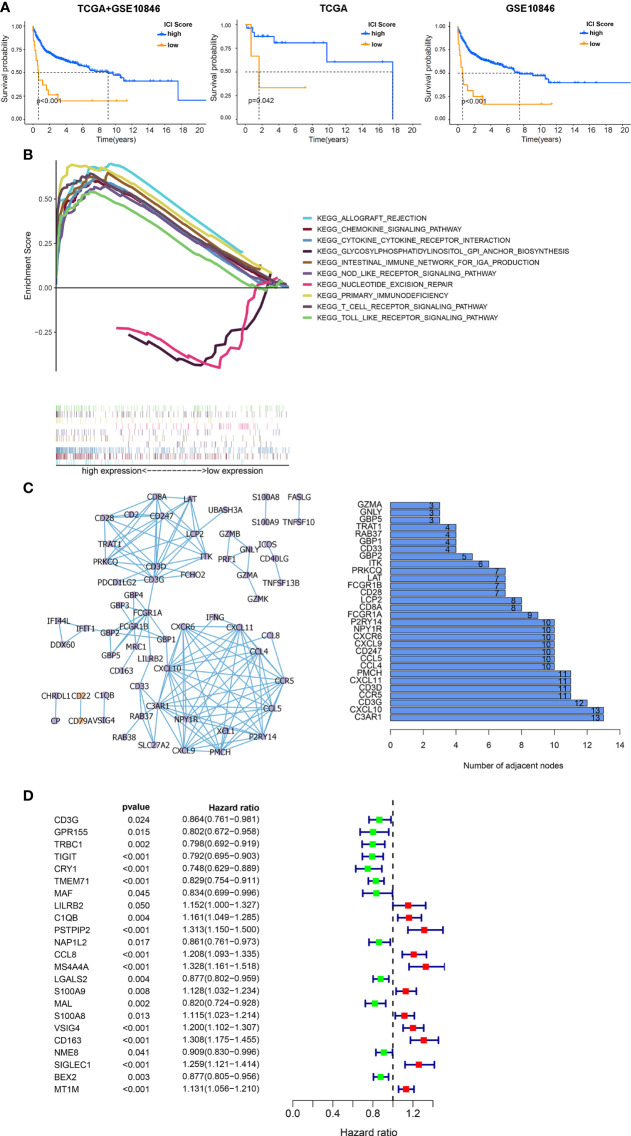
Functional analysis of DEGs among ICI clusters. **(A)** Kaplan–Meier survival analysis according to ICI scores. Left: the combination of the GSE10846 and TCGA databases. Middle: TCGA database. Right: GSE10846 dataset. **(B)** Multiple GSEAs were used to annotate the KEGG pathway enriched in high- and low-ICI score subgroups. **(C)** PPI network of hub genes (left: PPI network, purple: upregulated genes in tumor, orange: downregulated genes in tumor, right: bar plot demonstrating the number of adjacent nodes in the top 30 hub genes.). **(D)** Cox regressions were applied to detect the hazard ratios (HRs) of survival time. The confidence intervals are shown as the length of the line. Lines crossing HR = 1.0 indicates their insignificance.

Given that heterogeneity in DLBCL is associated with cell types, and the prognostic role of TME in DLBCL has been shown previously, we speculated that patients’ outcome was entwined with the heterogeneity of ICI. In this study, each patient was scored according to the expression of ICI signature genes A and B, and a final ICI score was obtained by subtracting the B score from the A score. Overall survival time was determined by dividing the patients into high- or low-ICI score groups based on median scores. The Kaplan–Meier plot indicated that patients with ICI low scores exhibit conspicuous prognosis advantages in GSE10864 (*P <* 0.01), TCGA datasets (*P =* 0.042), and the mixture of these two datasets (*P <* 0.01, **Figure 4A**). These results suggested that peritumor immune cell infiltration correlates with clinical features and prognosis.

To explore the biological features of the ICI score in depth, functional enrichment studies were conducted. The multiple GSEA suggested that genes in high-ICI score groups were mainly concentrated in immune-related pathways, including the chemokine signaling pathway, cytokine–cytokine receptor interaction, nod-like receptor signaling, primary immunodeficiency, and T-cell receptor signaling pathway (**Figure 4B**). Subsequently, all ICI-related DEGs were employed to predict protein interactions through the STRING website and 63 genes indicated as high confidence (0.9). The next analysis was undertaken *via* Cytoscape, and the bar plots displayed the top 30 genes ranked by the number of interaction nodes (**Figure 4C**). To better investigate the prognostic role of these hub genes, DEGs’ association with survival time was assessed using univariate Cox regression analyses performed on 312 samples in GSE10846 datasets, eliminating 108 cases with no data on DEGs. Proline–serine–threonine phosphatase-interacting protein 2 (PSTPIP2), CCL8, membrane spanning 4-domains A4A (MS4A4A), V-set and immunoglobulin domain containing 4 (VSIG4), CD163, sialic acid binding Ig-like lectin 1 (SIGLEC1), and metallothionein 1M (MT1M) were all found to be risky genes related to OS with HR > 1 and P < 0.01 (**Figure 4D**).

### Relevance of hub genes’ expression to clinical features and prognosis

**Figure 5:**
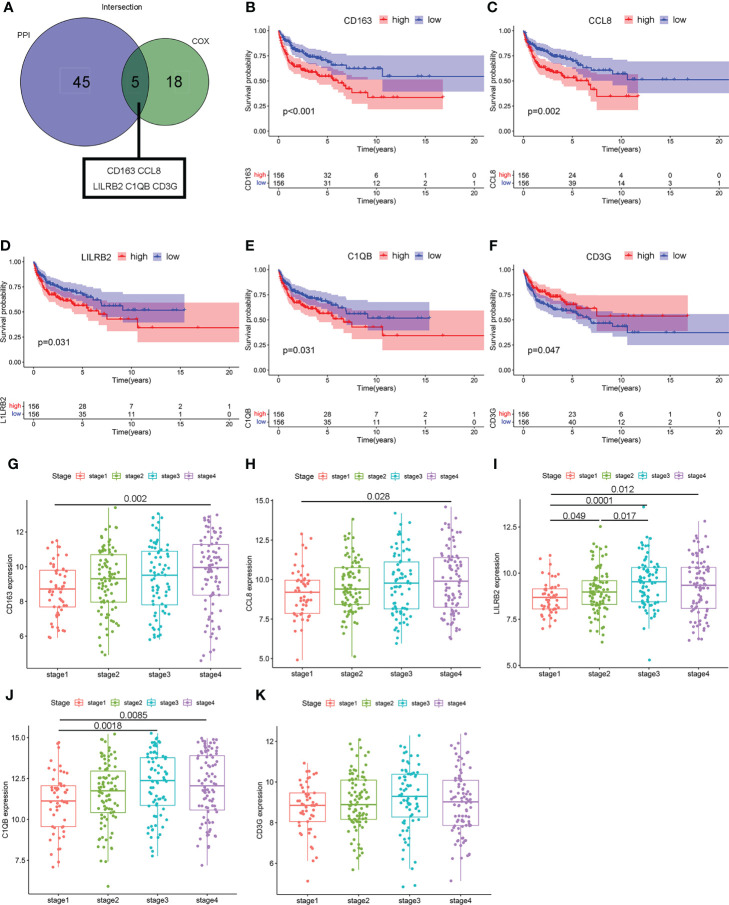
The relevance of hub genes to clinicopathologic features and over survival. **(A)** The Venn diagram indicating intersections of leading 50 nodes in PPI and top significant factors in univariate COX. **(B–F)** Kaplan–Meier curves validating survival difference between the high and low expressions of these five hub genes (CCL8, C1QB, CD3G, CD163, and LILRB2). **(G–K)** Scatter plot showing the association between the expression of five hub genes and clinical stages.

To minimize the systematic error from group classifications, the overlap of genes was assessed among the top 50 genes with leading nodes in the PPI network and 23 factor genes ranked by the univariate COX regression. Consequently, five genes (complement C1q B chain (C1QB), CCL8, CD3G, CD163, and leukocyte immunoglobulin-like receptor B2 (LILRB2)) were indicated (**Figure 5A**). A prognostic assessment was performed to investigate their potentials as prognostic factors in 312 cases from GSE10846, removing samples without genetic data. Lower expressions of CD163 (*P <* 0.001), CCL8 (*P =* 0.002), LILRB2 (*P =* 0.031), and C1QB (*P =* 0.031) showed significantly higher OS rates, whereas CD3G (*P =* 0.047) was negatively correlated with the overall survival time (**Figures 5B–F**). Moreover, four of these five genes, other than CD3G (*P >* 0.05), showed a significant increase in clinical stage 4 compared with stage 1 (*P =* 0.002, *P =* 0.028, *P =* 0.012, and *P =* 0.0085, **Figures 5G–K**). The above results suggested that the expression of these hub genes was associated with prognosis of DLBCL patients.

### Validation of clinical relevance of CCL8+CD163+ cells in DLBCL

**Figure 6:**
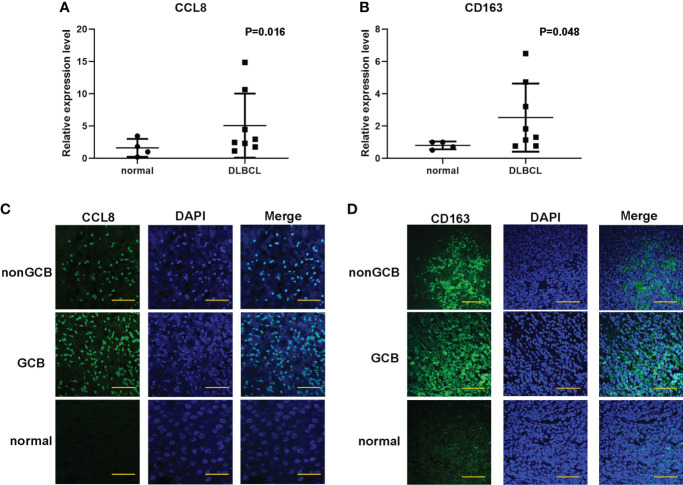
Validation of the expression of CCL8 and CD163 levels in DLBCL tissues and paracancer tissues. **(A, B)** q-PCR analysis for the mRNA expression of CCL8 and CD163 in DLBCL tissues and paracancer tissues. Statistical test was the multiple t test. **(C, D)** Tissue immunofluorescence imaging analysis to detect the amounts of CCL8 and CD163 in paraffin sections. Scale bars: 100 μm.

Based on the abovementioned results, the mRNA expression of these five hub genes was analyzed in four cases of normal lymph node tissues and eight cases of DLBCL tissues to quantify. The data showed that the expression levels of CCL8 (*P =* 0.016) and CD163 (*P =* 0.048) were obviously higher in DLBCL tissues compared to normal tissues, while there was no significant difference in LILRB2 (*P =* 0.788), C1QB (*P =* 0.556), and CD3G (*P =* 0.250) (**Figures 6A, B**, **Supplemental Figures 2A–C**). DLBCL is usually classified into germinal center B cell (GCB) type with better prognosis or nongerminal center B cell (non-GCB) type with worse prognosis based on the immunohistochemical expression. Immunofluorescence results showed similar results, with higher expressions of CCL8 and CD163 in non-GCB compared to GCB DLBCL (**Figures 6C, D**). These results further suggest the important prognostic values of CCL8 and CD163 in DLBCL specimens.

### Correlation of CCL8 with immune cell infiltration and clinical features

**Figure 7:**
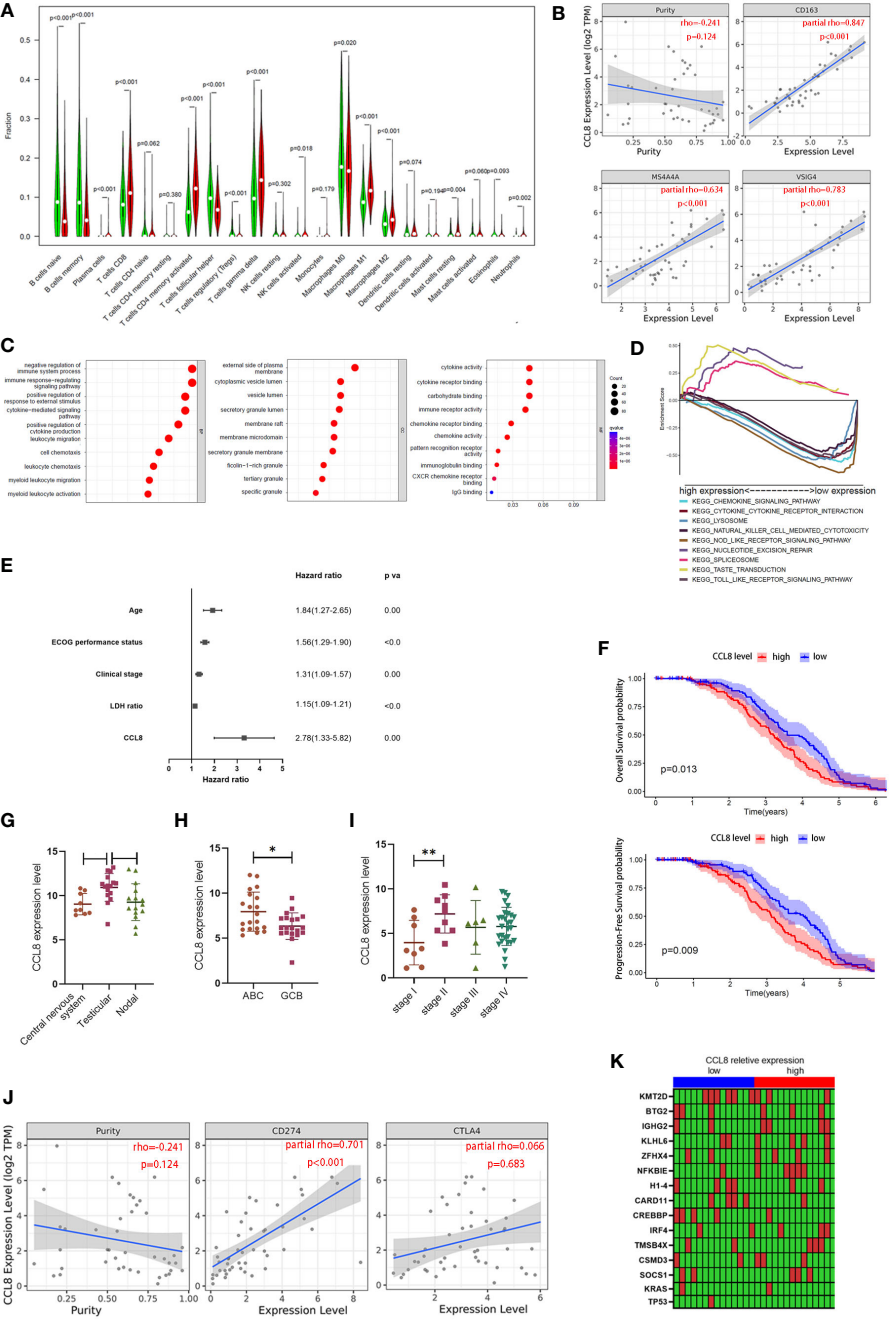
Association between CCL8 expression, M2 macrophage proportion, and clinical features. **(A)** The ratio differences of 21 kinds of immune cells between DLBCL samples with low or high CCL8 expression in TCGA and GEO databases. Statistical test was the Wilcoxon test. **(B)** The correlation between CCL8 expression and M2 macrophage cell markers in DLBCL *via* the TIMER website. Statistical test was the Spearman rank correlation test. **(C)** Bubble plot for GO enrichment of CCL8-related DEGs. Statistical test was the nonparametric test (the x-axis represents the gene, and the y-axis represents the description of the pathway; the circled area is proportional to the number of genes assigned to the term, and the color corresponds to the adjusted P-value; CC: cellular components; BP: biological process; MF: molecular function). **(D)** Multiple GSEA was used to annotate the KEGG pathway for CCL8-related DEGs. **(E)** Forest plot showing the hazard ratios of the clinical features and the CCL8 expression level to the overall survival benefits in GSE10846. **(F)** Kaplan–Meier curves validating OS and PFS in GSE136971. **(G–I)** Relevance between CCL8 expression and clinical features in GEO datasets (GSE10524, GSE64555, and GSE114175). Statistical tests were Mann–Whitney U tests and ANOVA tests. **(J)** Relationships between CCL8 expression and immune checkpoints in DLBCL. Statistical test was Spearman rank correlation test. **(K)** Heatmap of the relationship between mutant genes and CCL8 expression. Statistical test was the Mann–Whitney U test. *P<0.05, **P<0.01.

Chemokine CCL8, which is a member of a conserved chemokine cluster, plays a role in cancer metastasis by modulation of the tumor-promoting activity in breast cancer including recruiting M2 macrophages. Little is known about its biological function in other cancers, for example, in DLBCL. To further uncover the association between CCL8 expression and the immune microenvironment, the CIBERSORT algorithm was used to determine the proportion of tumor-infiltrating immune cell subsets in 420 cases in the GSE10846 database and 29 cases in TCGA database. Twenty-one kinds of immune cell profiles were evaluated in the high- and low-CCL8 expression groups compared with the median level of CCL8. The results showed that a total of 15 kinds of TICs were associated with the expression of CCL8 including M2 macrophages (**Figure 7A**).

According to previous findings that CD163, the cell marker of M2 macrophages, is one of the hub genes, we explored the link between M2 macrophage featured genes (CD163, MS4A4A, and VSIG4) and CCL8 expression in DLBCL *via* the TIMER database. We found that all of these three markers were positively correlated with CCL8 expression (R > 0, *P <* 0.001, **Figure 7B**). Given that the expression level of CCL8 was negatively associated with the survival time and clinical stages in DLBCL patients, GO enrichment analysis and GSEA were used to investigate the underlying interplay of DESs between high- and low-CCL8 expression groups. It can be inferred that immune-related processes and secretory granule membranes, which were associated with cytokine and chemokine activity, were highly associated with CCL8-related DEGs (**Figure 7C**). A multiple-GSEA investigation also indicated that DEGs in the low-CCL8 expression group were enriched in the chemokine signaling pathway and the cytokine–cytokine receptor interaction pathway (**Figure 7D**).

Next, we probed into the CCL8 expression in other independent cohorts to find its potential as a prognostic factor. Uni- and multi-Cox regression analyses indicated that the age, ECOG performance status, clinical stages, LDH ratio, and CCL8 expression level were independent prognostic factors for predicting survival in DLBCL patients from the GSE10846 dataset (**Supplemental Tables 3** and **4**, **Figure 7E**). In GSE136971, the CCL8 level was negatively associated with overall patient survival (*P =* 0.013) and progression-free survival probability (*P =* 0.009, **Figure 7F**). The site of onset is also associated with CCL8 expression. As shown in GSE10524, testicular DLBCL contained more CCL8 than the central nervous system (*P <* 0.05) and nodal DLBCL (*P =* 0.056, **Figure 7G**). The expression of CCL8 in activated B-cell-like (ABC) DLBCL, a subtype with worse prognosis, was significantly lower than that in germinal-center B cell-like (GCB) DLBCL in GSE64555 (*P <* 0.05, **Figure 7H**). Patients in stage II also had a greater amount of CCL8 than in stage I according to the analysis in GSE114175 (P < 0.01, **Figure 7I**). Moreover, CCL8 had a significant positive correlation with PD-L1 (rho = 0.701, P < 0.001) but not CTLA4 (rho = 0.066, P > 0.05) in DLBCL *via* TIMER website analysis (**Figure 7J**). Therefore, CCL8 might be used as a diagnostic biomarker in DLBCL and was highly associated with M2 macrophage content.

The random forest algorithm was used to explore the importance of gene mutation related to CCL8 expression in DLBCL. A total of 2,156 gene mutations were identified in TCGA cohort, including TP53 and KRAS (**Supplemental Table 5**). Genes with greater than five mutations in 27 cases were selected for further analysis. The detailed mutation types of these genes are shown in **Supplemental Figure 3**. Furthermore, CREBBP mutation was correlated with CCL8 expression (*P* = 0.013), composed of missense mutations and in-frame deletions (**Figure 7K**).

## Discussion

**Figure 8:**
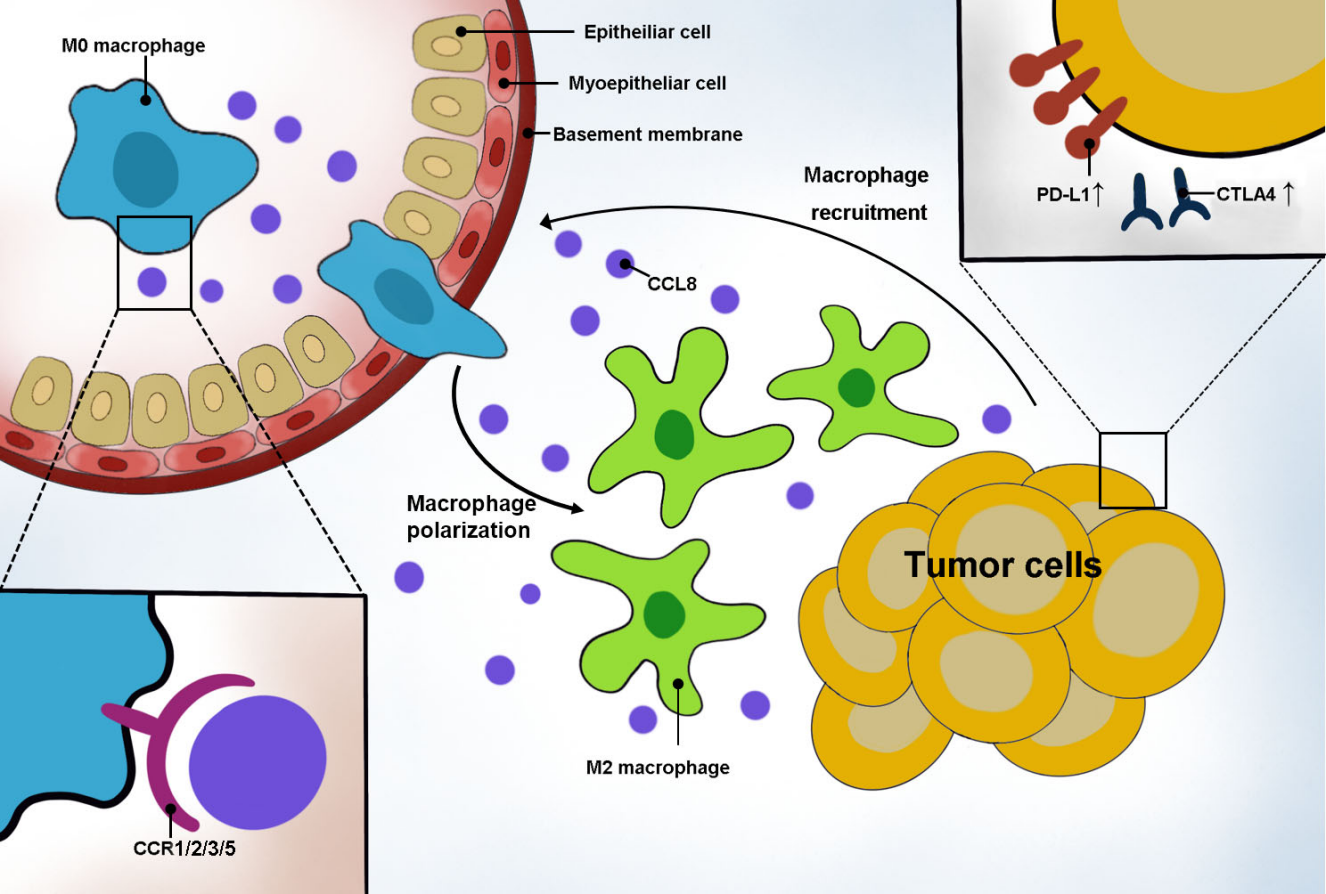
Schematic representation of CCL8’s role in DLBCL. Tumor cells secreted CCL8 to recruit macrophage moving around the tumor by binding with the CCR family and promoting macrophage polarization to the M2 type. In addition, highly CCL8-secreting tumor cells had an elevated expression of PD-L1 and CTLA4.

DLBCL is an aggressive lymphoma composed of malignant B cells, and its pathogenesis is tied to immune cell constitution (16). Recently, molecular research into the TME of DLBCL has attempted to identify new specific prognostic and risk stratification biomarkers to predict patients’ outcome. In this study, we aimed to examine the relationship between ICI and prognosis in DLBCL and to identify the ICI-related genes that contribute to the survival of patients in GEO and TCGA datasets. CCL8 was identified to be involved in the immune response by interacting with M2 macrophages. In addition, bioinformatic analysis provided convincing evidence of a strong association between CCL8 and clinical features in DLBCL (**Figure 8**).

It was demonstrated that the role of the TME is dependent on the interaction between tumor cells and peritumor immune cells, such as T cells, B cells, NK cells, and macrophages, combined with stromal elements (17). Autio et al. have suggested that poor survival is associated with a high proportion of positive T-cell checkpoints through immunophenotyping in DLBCL (16). Benedetta Apollonio’s discussion of the immune-stromal microenvironment in B-cell lymphoma openly acknowledges that nonhematopoietic stromal cells can modulate antitumor immunity by constituting a barrier to infiltrating T cells (18). In chronic lymphocytic leukemia, splenic stromal cells coevolve with the disease by producing the pro-B cell cytokine CXCL13 (19). The infiltration of stromal cells also has positive prognostic impact on DLBCL outcomes confirmed by Dubois (20) and Schmitz et al. (21), respectively. Based on these studies, we conducted a systematic analysis of the transcriptional DLBCL data in the GEO and TCGA datasets, which suggested that the stromal score in the TME was conducive to the patients’ prognosis. These results emphasize the importance of exploring the effects of peritumor immune cells on tumor cells, which can propose new ideas for immunotherapy in DLBCL.

DLBCL is a disease in which malignant B cells accumulate in nodal and extranodal sites, and the number of extranodal involvement sites correlates with the clinical stage. Our results showed a positive correlation between the stromal score and the number of extranodal involvement sites. The work of Antonio Giovanni Solimando et al. sums up the pivotal role of a cancer-friendly environment in patients with both nodal and extranodal site invasion, stating that single cells were able to reside and survive in nodal and extranodal sites as stromal cells and vessels in the TME provide nutrients and oxygen for them and become a source of residual disease and recurrence (22). In addition, the immune scores were elevated from clinical stages 1 to 3 but decreased in stage 4, which indicates that tumor progression is accompanied by an enhanced tumor immune response but is gradually exhausted with tumor spreading. Similar to our results, Marie Tosolini distinguished the cancer immune cell activity in B-NHL into four stages based on differences in gene expression of each DLBCL sample in public databases, revealing that the higher the stage, the stronger the immune response and the more the immune escape, which implies poorer prognosis (1).

Interestingly, the ICI score derived by the PCA algorithm, which is the score of ICI A feature genes minus that of ICI B, was positively correlated with the outcome of patients. This may be on account of the independent effect of each immune cell on clinical features and survival. Moreover, the ICI A group was more involved in immune-related pathways such as the chemokine signaling pathway. Previous studies have shown that DLBCL cells express the ligand C–C motif chemokines to suppress antitumor immune responses through recruiting immune cells that secrete the tumor-promoting factors (23, 24).

Next, we found that the prognosis of patients was more closely related to γδT cells and macrophages by analyzing immune cells within different ICI subgroups and gene subgroups. A study carried out by Jung Hyun Her revealed that γδ T cells that express the FcγRIIIa receptor can be effector cells for tafasitamab, an Fc-modified monoclonal antibody, thus inducing antibody-dependent cellular cytotoxicity against a range of NHL cell lines (25). Further, some studies claimed that macrophage polarization in DLBCL was associated with CREBBP/EP300 mutations and in turn promotes tumor progression (26).

From the results obtained by overlapping the PPI and prognosis-related genes detected through COX analyses, we unveiled that the core differential genes include CD163 and CCL8. CD163 is widely accepted as the surface marker of M2 macrophages, which play an important role in B-cell lymphoma by restraining host antitumor immune effector responses (27, 28). The high expression of CD163 was notably related to poor survival and clinical characteristics (age, ECOG, number of extranodal sites, and stage). M2 macrophages express inflammation-suppressing factors that serve to suppress tumor immune responses and promote tumor growth (29). A training set of 132 cases showed that DLBCL had a different number of M2 macrophages, and the high infiltration of CD163 and pentraxin 3 (PTX3) was associated with a low survival rate (30).

Reiser et al. discovered that the underlying mechanism by which M2 macrophages enhance tumorigenesis is through secreting proinflammatory cytokines, which are influenced by CD8+ T cells (31). However, the regulation mechanism between M2 macrophages and tumor cells has not been illustrated clearly yet. Some studies suggest that it may be linked to cytokines and chemokines secreted by tumor cells (32). By interacting with homologous receptors and acting as the MAPK/ERK pathway, cytokines and chemokines affect immune cell chemotaxis and activation, angiogenesis, and tumor cell metastasis (33). In glioblastoma, tumor cells recruit M2 macrophages in response to CCL2 (34).

CCL8, a chemokine, is shown to be negatively associated with patient prognosis in glioma, colorectal cancer, kidney cancer, uroepithelial cancer, testicular cancer, breast cancer, and endometrial cancer and positively associated with patient prognosis in thyroid cancer, head and neck tumors, and ovarian cancer in The Human Protein Atlas database. CCL8 is a ligand for CCR2 and activates CCR1, CCR3, and CCR5. It recruits TAM to the tumor perimeter by binding to CCR1, contributing to the increased susceptibility of tumors to metastasize to the liver. After binding to CCR2, it induces tumor cells to metastasize to the bone through TAM-mediated tumor angiogenesis. In addition, it is also able to bind to CCR3, thus promoting tumor angiogenesis (35). In breast cancer, CCL8 enhances tumor cell activity and contributes to tumor metastasis by regulating the TME, and the pro-cancer effect of CCL8 is inhibited upon macrophage depletion, suggesting that CCL8 may promote tumor progression by recruiting macrophages (36). In cervical cancer, CCL8 causes the recruitment of TAM through interactions with ZEB1 in hypoxic cancer cells (37).

A study carried out by Hiroki Kobayashi revealed that fibroblasts promote macrophage chemotaxis by enhancing nuclear factor-κB-IL34/CCL8 signaling in colorectal carcinogenesis. Despite most studies focusing on epithelial tumors, little is known about the role of CCL8 on macrophages in sarcoma. The research on 1,242 sarcoma specimens indicated that macrophages outnumbered tumor-infiltrating lymphocytes and M2 macrophages were more prominent than M1 macrophages (38). In Ewing sarcoma, USP6 induced macrophage chemotaxis by enhancing the production of CXCL10 and CCL5 (39). We verified the positive correlation of CCL8 expression with M2 macrophages by CIBERSORT analysis of the GSE10846 dataset and online analysis of the TIMER dataset. Combining the results of previous studies, it is likely that in DLBCL, CCL8 is able to recruit M2 macrophages to promote tumor metastasis.

Further investigations should be conducted to clarify the relationship between tumor-infiltrating macrophages and CCL8 expression, especially in tumor metastasis-related pathways and TAM-dependent angiogenesis. It is also worth investigating which ligands CCL8 binds to, including CCR1, CCR2, CCR3, and CCR5, and thus how it affects the downstream signaling pathways that lead to changes in the biological behavior of tumor cells.

## Conclusions

In summary, we found a significant correlation between patients’ outcome and peritumor macrophages through bioinformatics data mining of TCGA and GEO datasets. We also established the association of CCL8 with CD163, which is the surface marker of M2 macrophages. We speculated that CCL8 may play a key role in immune escape of DLBCL by interacting with M2 macrophages.

## Data availability statement

The original contributions presented in the study are included in the article/**Supplementary Material**. Further inquiries can be directed to the corresponding authors.

## Ethics statement

Written informed consent was obtained from the individual(s) for the publication of any potentially identifiable images or data included in this article. The project was approved by the ethics committee of the Second Affiliated Hospital of Soochow University (Ethics ID: JD-HG-2022-37).

## Author contributions

XL downloaded data from the public database and performed bioinformatics analysis. KZ performed qPCR and immunofluorescence analysis. JX and LS organized figures and results. HN and ZC provided tissues. JW and YZ designed the study and wrote the paper. All authors contributed to the article and approved the submitted version.

## Funding

This study was supported by grants from the Advance Research Program of the Second Affiliated Hospital of Soochow University (SDFEYQN2001), the Youth Research Programs of Health Commission Foundation of Suzhou (KJXW2021015), the Suzhou Science and Technology Development Program (SYS2020146), and the Joint Research Center for Genomic Resources 2017B01012 (2021K003).

## Acknowledgments

Tissue samples were provided by the tissue bank of the Second Affiliated Hospital of Soochow University (Suzhou, China) with the approval of the Ethics Committee of Soochow University.

## Conflict of interest

The authors declare that the research was conducted in the absence of any commercial or financial relationships that could be construed as a potential conflict of interest.

## Publisher’s note

All claims expressed in this article are solely those of the authors and do not necessarily represent those of their affiliated organizations, or those of the publisher, the editors and the reviewers. Any product that may be evaluated in this article, or claim that may be made by its manufacturer, is not guaranteed or endorsed by the publisher.

## References

[1] Tosolini M Algans C Pont F Ycart B Fournie JJ. Large-Scale microarray profiling reveals four stages of immune escape in non-Hodgkin lymphomas. Oncoimmunology (2016) 5(7):e1188246. doi: 10.1080/2162402X.2016.1188246 27622044 PMC5006906

[2] Pi M Kuang H Yue C Yang Q Wu A Li Y. Targeting metabolism to overcome cancer drug resistance: A promising therapeutic strategy for diffuse large b cell lymphoma. Drug Resist Update (2022) 61:100822. doi: 10.1016/j.drup.2022.100822 35257981

[3] Aghanejad A Bonab SF Sepehri M Haghighi FS Tarighatnia A Kreiter C. A review on targeting tumor microenvironment: The main paradigm shift in the mAb-based immunotherapy of solid tumors. Int J Biol Macromol (2022) 207:592–610. doi: 10.1016/j.ijbiomac.2022.03.057 35296439

[4] Kline J Godfrey J Ansell SM. The immune landscape and response to immune checkpoint blockade therapy in lymphoma. Blood (2020) 135(8):523–33. doi: 10.1182/blood.2019000847 31790142

[5] Hopken UE Rehm A. Targeting the tumor microenvironment of leukemia and lymphoma. Trends Cancer (2019) 5(6):351–64. doi: 10.1016/j.trecan.2019.05.001 31208697

[6] Kumar D Xu ML. Microenvironment cell contribution to lymphoma immunity. Front Oncol (2018) 8:288 [published Online First: 2018/08/14. doi: 10.3389/fonc.2018.00288 30101129 PMC6073855

[7] Niu Y Chen J Qiao Y. Epigenetic modifications in tumor-associated macrophages: A new perspective for an old foe. Front Immunol (2022) 13:836223 [published Online First: 2022/02/11. doi: 10.3389/fimmu.2022.836223 35140725 PMC8818998

[8] Hornburg M Desbois M Lu S Guan Y Lo AA Kaufman S. Single-cell dissection of cellular components and interactions shaping the tumor immune phenotypes in ovarian cancer. Cancer Cell (2021) 39(7):928–44.e6. doi: 10.1016/j.ccell.2021.04.004 33961783

[9] Grunwald BT Devisme A Andrieux G Vyas F Aliar K McCloskey CW. Spatially confined sub-tumor microenvironments in pancreatic cancer. Cell (2021) 184(22):5577–92.e18. doi: 10.1016/j.cell.2021.09.022 34644529

[10] Schreiber RD Old LJ Smyth MJ. Cancer immunoediting: integrating immunity's roles in cancer suppression and promotion. Science (2011) 331(6024):1565–70. doi: 10.1126/science.1203486 21436444

[11] O'Connor T Zhou X Kosla J Adili A Garcia Beccaria M Kotsiliti E. Age-related gliosis promotes central nervous system lymphoma through CCL19-mediated tumor cell retention. Cancer Cell (2019) 36(3):250–67.e9. doi: 10.1016/j.ccell.2019.08.001 31526758

[12] Wang WG Jiang XN Sheng D Sun CB Lee J Zhou XY. PD-L1 over-expression is driven by b-cell receptor signaling in diffuse large b-cell lymphoma. Lab Invest (2019) 99(10):1418–27. doi: 10.1038/s41374-019-0262-5 31197205

[13] Xu-Monette ZY Xiao M Au Q Padmanabhan R Xu B Hoe N. Immune profiling and quantitative analysis decipher the clinical role of immune-checkpoint expression in the tumor immune microenvironment of DLBCL. Cancer Immunol Res (2019) 7(4):644–57. doi: 10.1158/2326-6066.CIR-18-0439 30745366

[14] Zhu W Xie L Han J Guo X. The application of deep learning in cancer prognosis prediction. Cancers (Basel) (2020) 12(3):603. doi: 10.3390/cancers12030603 PMC7139576 32150991

[15] Yoshihara K Shahmoradgoli M Martinez E Vegesna R Kim H Torres-Garcia W. Inferring tumour purity and stromal and immune cell admixture from expression data. Nat Commun (2013) 4:2612. doi: 10.1038/ncomms3612 24113773 PMC3826632

[16] Autio M Leivonen SK Bruck O Mustjoki S Meszaros Jorgensen J Karjalainen-Lindsberg ML. Immune cell constitution in the tumor microenvironment predicts the outcome in diffuse large b-cell lymphoma. Haematologica (2021) 106(3):718–29. doi: 10.3324/haematol.2019.243626 PMC7927991 32079690

[17] Zhang X Shi M Chen T Zhang B. Characterization of the immune cell infiltration landscape in head and neck squamous cell carcinoma to aid immunotherapy. Mol Ther Nucleic Acids (2020) 22:298–309. doi: 10.1016/j.omtn.2020.08.030 33230435 PMC7522342

[18] Apollonio B Ioannou N Papazoglou D Ramsay AG. Understanding the immune-stroma microenvironment in b cell malignancies for effective immunotherapy. Front Oncol (2021) 11:626818 [published Online First: 2021/04/13. doi: 10.3389/fonc.2021.626818 33842331 PMC8027510

[19] Farinello D Wozinska M Lenti E Genovese L Bianchessi S Migliori E. A retinoic acid-dependent stroma-leukemia crosstalk promotes chronic lymphocytic leukemia progression. Nat Commun (2018) 9(1):1787. doi: 10.1038/s41467-018-04150-7 29725010 PMC5934403

[20] Dubois S Tesson B Mareschal S Viailly PJ Bohers E Ruminy P. Refining diffuse large b-cell lymphoma subgroups using integrated analysis of molecular profiles. EBioMedicine (2019) 48:58–69. doi: 10.1016/j.ebiom.2019.09.034 31648986 PMC6838437

[21] Schmitz R Wright GW Huang DW Johnson CA Phelan JD Wang JQ. Genetics and pathogenesis of diffuse Large b-cell lymphoma. N Engl J Med (2018) 378(15):1396–407. doi: 10.1056/NEJMoa1801445 PMC6010183 29641966

[22] Solimando AG Annese T Tamma R Ingravallo G Maiorano E Vacca A. New insights into diffuse Large b-cell lymphoma pathobiology. Cancers (Basel) (2020) 12(7):1869. doi: 10.3390/cancers12071869 PMC7408689 32664527

[23] Higuchi T Matsuo K Hashida Y Kitahata K Ujihara T Taniguchi A. Epstein-Barr Virus-positive pyothorax-associated lymphoma expresses CCL17 and CCL22 chemokines that attract CCR4-expressing regulatory T cells. Cancer Lett (2019) 453:184–92. doi: 10.1016/j.canlet.2019.03.053 30953706

[24] Manfroi B McKee T Mayol JF Tabruyn S Moret S Villiers C. CXCL-8/IL8 produced by diffuse Large b-cell lymphomas recruits neutrophils expressing a proliferation-inducing ligand APRIL. Cancer Res (2017) 77(5):1097–107. doi: 10.1158/0008-5472.CAN-16-0786 27923834

[25] Her JH Pretscher D Patra-Kneuer M Schanzer J Cho SY Hwang YK. Tafasitamab mediates killing of b-cell non-hodgkin's lymphoma in combination with gammadelta T cell or allogeneic NK cell therapy. Cancer Immunol Immunother (2022) CII. doi: 10.1007/s00262-022-03165-w PMC9519642 35348812

[26] Huang YH Cai K Xu PP Wang L Huang CX Fang Y. CREBBP/EP300 mutations promoted tumor progression in diffuse large b-cell lymphoma through altering tumor-associated macrophage polarization *via* FBXW7-NOTCH-CCL2/CSF1 axis. Signal Transduct Target Ther (2021) 6(1):10. doi: 10.1038/s41392-020-00437-8 33431788 PMC7801454

[27] Lin Y Gustafson MP Bulur PA Gastineau DA Witzig TE Dietz AB. Immunosuppressive CD14+HLA-DR(low)/- monocytes in b-cell non-Hodgkin lymphoma. Blood (2011) 117(3):872–81. doi: 10.1182/blood-2010-05-283820 PMC3035079 21063024

[28] McKee SJ Tuong ZK Kobayashi T Doff BL Soon MS Nissen M. B cell lymphoma progression promotes the accumulation of circulating Ly6Clo monocytes with immunosuppressive activity. Oncoimmunology (2018) 7(2):e1393599. doi: 10.1080/2162402X.2017.1393599 29308328 PMC5749670

[29] Cassetta L Kitamura T. Macrophage targeting: opening new possibilities for cancer immunotherapy. Immunology (2018) 155(3):285–93. doi: 10.1111/imm.12976 PMC6187207 29963704

[30] Carreras J Kikuti YY Hiraiwa S Miyaoka M Tomita S Ikoma H. High PTX3 expression is associated with a poor prognosis in diffuse large b-cell lymphoma. Cancer Sci (2022) 113(1):334–48. doi: 10.1111/cas.15179 PMC8748251 34706126

[31] Reiser J Banerjee A. Effector, memory, and dysfunctional CD8(+) T cell fates in the antitumor immune response. J Immunol Res (2016) 2016:8941260. doi: 10.1155/2016/8941260 27314056 PMC4893440

[32] Korbecki J Grochans S Gutowska I Barczak K Baranowska-Bosiacka I. CC chemokines in a tumor: A review of pro-cancer and anti-cancer properties of receptors CCR5, CCR6, CCR7, CCR8, CCR9, and CCR10 ligands. Int J Mol Sci (2020) 21(20):7619. doi: 10.3390/ijms21207619 PMC7590012 33076281

[33] Mollica Poeta V Massara M Capucetti A Bonecchi R. Chemokines and chemokine receptors: New targets for cancer immunotherapy. Front Immunol (2019) 10:379 [published Online First: 2019/03/22. doi: 10.3389/fimmu.2019.00379 30894861 PMC6414456

[34] Takenaka MC Gabriely G Rothhammer V Mascanfroni ID Wheeler MA Chao CC. Control of tumor-associated macrophages and T cells in glioblastoma *via* AHR and CD39. Nat Neurosci (2019) 22(5):729–40. doi: 10.1038/s41593-019-0370-y PMC8052632 30962630

[35] Korbecki J Kojder K Siminska D Bohatyrewicz R Gutowska I Chlubek D. CC chemokines in a tumor: A review of pro-cancer and anti-cancer properties of the ligands of receptors CCR1, CCR2, CCR3, and CCR4. Int J Mol Sci (2020) 21(21):8412. doi: 10.3390/ijms21218412 PMC7665155 33182504

[36] Cassetta L Fragkogianni S Sims AH Swierczak A Forrester LM Zhang H. Human tumor-associated macrophage and monocyte transcriptional landscapes reveal cancer-specific reprogramming, biomarkers, and therapeutic targets. Cancer Cell (2019) 35(4):588–602.e10. doi: 10.1016/j.ccell.2019.02.009 30930117 PMC6472943

[37] Chen XJ Deng YR Wang ZC Wei WF Zhou CF Zhang YM. Hypoxia-induced ZEB1 promotes cervical cancer progression *via* CCL8-dependent tumour-associated macrophage recruitment. Cell Death Dis (2019) 10(7):508. doi: 10.1038/s41419-019-1748-1 31263103 PMC6602971

[38] Dancsok AR Gao D Lee AF Steigen SE Blay JY Thomas DM. Tumor-associated macrophages and macrophage-related immune checkpoint expression in sarcomas. Oncoimmunology (2020) 9(1):1747340. doi: 10.1080/2162402X.2020.1747340 32313727 PMC7153829

[39] Henrich IC Jain K Young R Quick L Lindsay JM Park DH. Ubiquitin-specific protease 6 functions as a tumor suppressor in Ewing sarcoma through immune activation. Cancer Res (2021) 81(8):2171–83. doi: 10.1158/0008-5472.CAN-20-1458 PMC8137534 33558334

