# Supplementary material for: CCL8 as a promising prognostic factor in diffuse large B-cell lymphoma via M2 macrophage interactions: A bioinformatic analysis of the tumor microenvironment
Source: Front Immunol. 2022 Aug 22;13:950213. doi: 10.3389/fimmu.2022.950213 (PMC9441746; doi:10.3389/fimmu.2022.950213)
Supplement: Supplementary file 1 [file DataSheet_1.docx]

**Supplemental Figures**

| **A** | **B** |
| --- | --- |
| 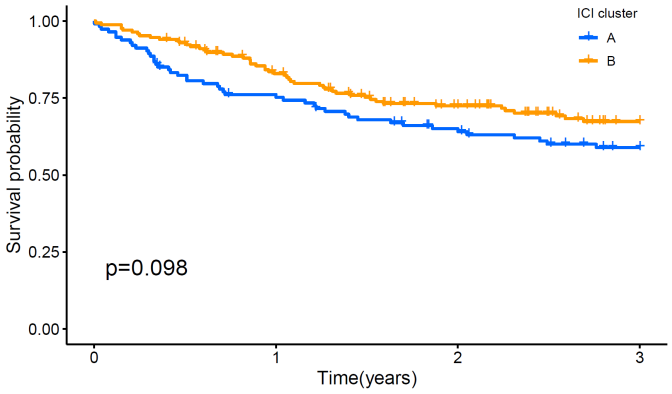 | 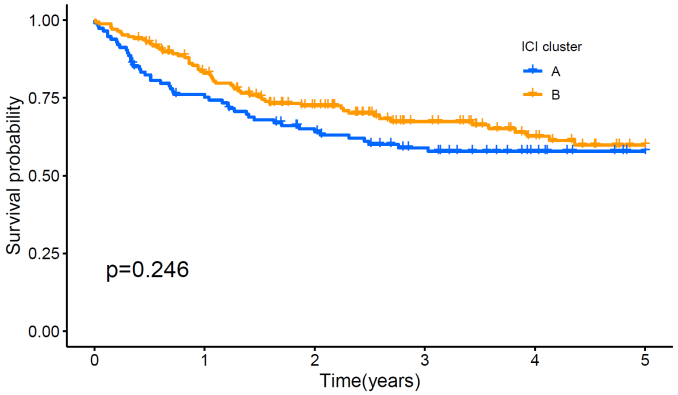 |
| **C** | **D** |
| 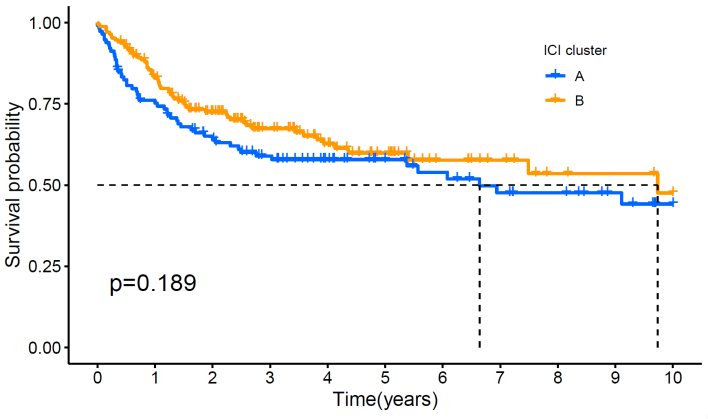 | 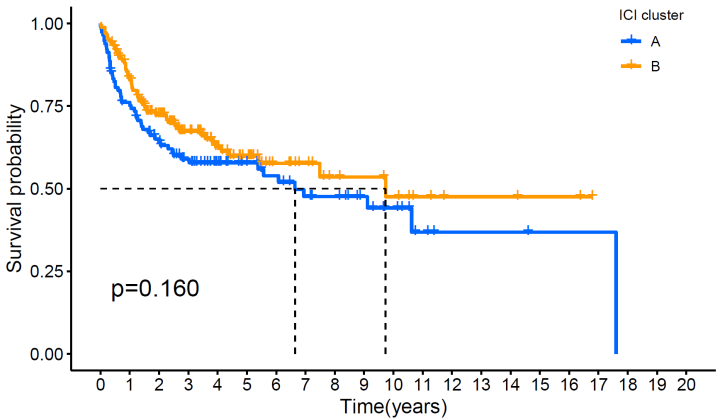 |

**Supplemental Figure 1.** Kaplan-Meier survival analysis according to ICI group. (A)3-year survival rate, (B) 5-year survival rate, (C)10-year survival rate, (D)20-year survival rate.

| A | B | C |
| --- | --- | --- |
|  |  |  |

**Supplemental Figure 2 Quantitative analysis for the mRNA expression of LILRB2 (A), C1QB (B) and CD3G (C) in DLBCL tissues and paracancer tissues.**


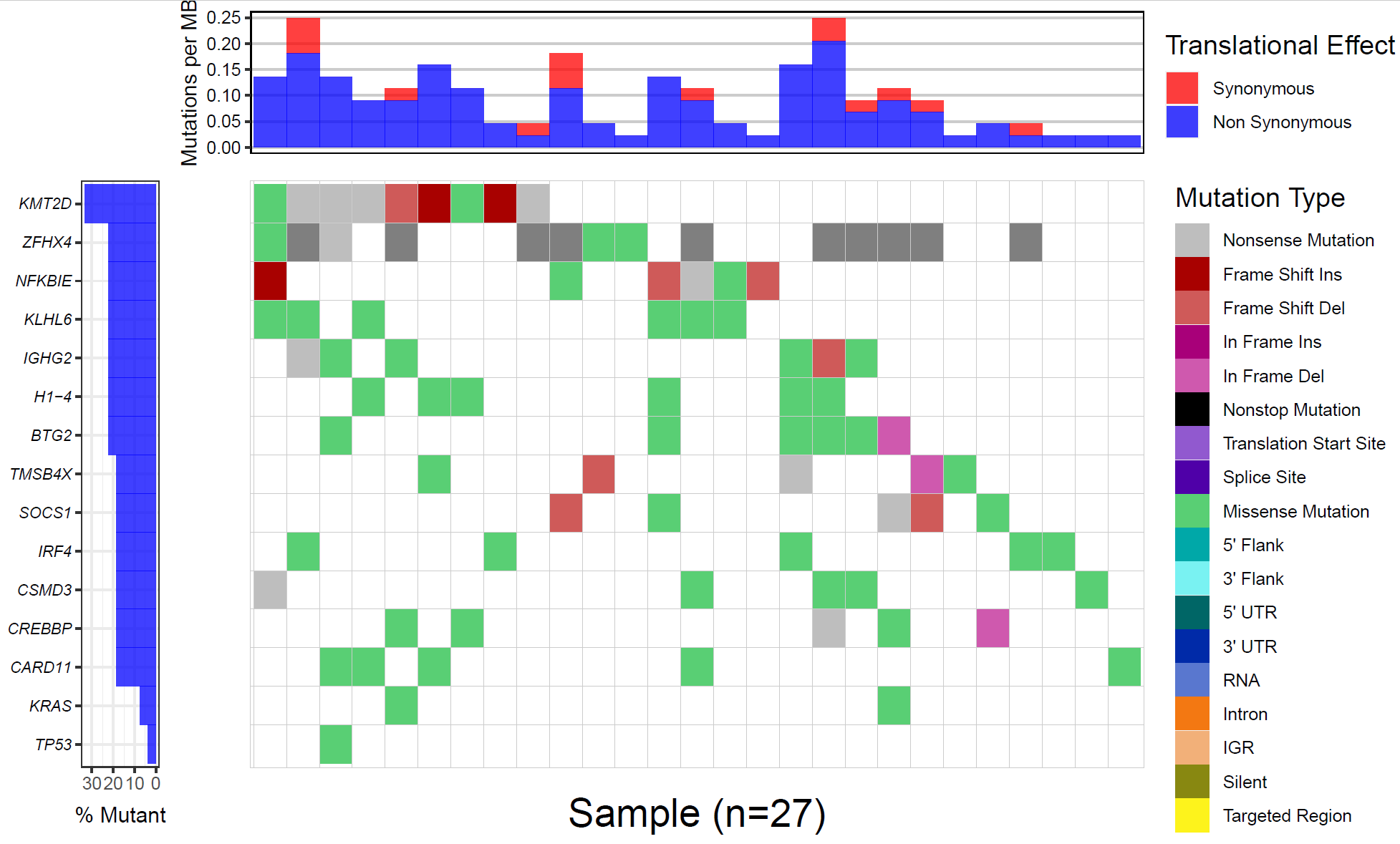


**Supplemental Figure 3 Waterfall plot of gene mutations in DLBCL patients from TCGA database.**
