# Supplementary material for: CCL8 as a promising prognostic factor in diffuse large B-cell lymphoma via M2 macrophage interactions: A bioinformatic analysis of the tumor microenvironment
Source: Front Immunol. 2022 Aug 22;13:950213. doi: 10.3389/fimmu.2022.950213 (PMC9441746; doi:10.3389/fimmu.2022.950213)
Supplement: Supplementary file 2 [file DataSheet_2.docx]

**Supplementary Table1 Primers**

| **Real-time PCR Primers** | | | |
| --- | --- | --- | --- |
| **Gene** | **Accession Number** | **Sequence forward primer (5´-3´)** | **Sequence reverse primer (5´-3´)** |
| C1QB | NP_000482 | TTCTGTGACTATGCCTACAACAC | GCCCAGTAGTGAGTTCTTGTC |
| CCL8 | NP_005614 | TGGAGAGCTACACAAGAATCACC | TGGTCCAGATGCTTCATGGAA |
| CD3G | NP_000064 | GGTTCGGTACTTCTGACTTGTG | TCAGTTAGGAAGCCGATCATCT |
| CD163 | NP_004235 | TTTGTCAACTTGAGTCCCTTCAC | TCCCGCTACACTTGTTTTCAC |
| LILRB2 | NP_001074447 | GCATCTTGGATTACACGGATACG | CTGACAGCCATATCGCCCTG |

**Supplementary Table 2 ICI-related DEGs**

| **id** | **logFC** | **AveExpr** | **t** | **P.Value** | **adj.P.Val** | **B** |
| --- | --- | --- | --- | --- | --- | --- |
| GZMA | 2.028124 | 11.10915 | 20.07919 | 1.52E-64 | 2.69E-60 | 135.8916 |
| THEMIS | 2.301111 | 7.581562 | 18.79584 | 1.24E-58 | 1.09E-54 | 122.4573 |
| CD8A | 1.794801 | 10.29272 | 18.75076 | 2.00E-58 | 1.17E-54 | 121.9869 |
| SH2D1A | 2.092282 | 8.157616 | 18.09581 | 1.99E-55 | 8.79E-52 | 115.1679 |
| CD3D | 1.893068 | 11.64563 | 18.05298 | 3.13E-55 | 1.10E-51 | 114.7232 |
| IFNG | 2.026421 | 8.75611 | 17.98884 | 6.14E-55 | 1.80E-51 | 114.0574 |
| CRTAM | 2.074154 | 8.840963 | 17.8715 | 2.10E-54 | 5.30E-51 | 112.8405 |
| GZMK | 2.091808 | 11.46624 | 17.74101 | 8.27E-54 | 1.82E-50 | 111.4887 |
| CD3G | 1.946553 | 8.536332 | 17.71116 | 1.13E-53 | 2.22E-50 | 111.1797 |
| GIMAP7 | 1.756304 | 10.00182 | 17.35476 | 4.71E-52 | 8.30E-49 | 107.4975 |
| GIMAP6 | 1.571624 | 9.596319 | 17.18426 | 2.79E-51 | 4.47E-48 | 105.7409 |
| ITM2A | 1.723887 | 10.29605 | 17.13023 | 4.89E-51 | 7.19E-48 | 105.185 |
| CXCR6 | 1.634518 | 8.478441 | 17.03817 | 1.28E-50 | 1.73E-47 | 104.2386 |
| TRAT1 | 2.099381 | 8.089731 | 16.57845 | 1.50E-48 | 1.89E-45 | 99.52907 |
| CST7 | 1.632668 | 10.38466 | 16.55243 | 1.97E-48 | 2.31E-45 | 99.26331 |
| FGL2 | 1.567466 | 10.56675 | 16.17795 | 9.33E-47 | 1.03E-43 | 95.45095 |
| **id** | **logFC** | **AveExpr** | **t** | **P.Value** | **adj.P.Val** | **B** |
| GIMAP4 | 1.545441 | 9.91993 | 16.06866 | 2.86E-46 | 2.97E-43 | 94.34249 |
| CD2 | 1.712422 | 11.15802 | 15.93128 | 1.17E-45 | 1.15E-42 | 92.95196 |
| GIMAP2 | 1.427532 | 10.2408 | 15.60324 | 3.33E-44 | 3.06E-41 | 89.64507 |
| GZMH | 1.938431 | 9.461484 | 15.5993 | 3.47E-44 | 3.06E-41 | 89.60543 |
| EOMES | 1.581384 | 10.2336 | 15.46722 | 1.33E-43 | 1.11E-40 | 88.2797 |
| UBASH3A | 2.399697 | 6.718676 | 15.31281 | 6.34E-43 | 5.09E-40 | 86.7341 |
| TNFSF10 | 1.182812 | 10.65321 | 15.00793 | 1.37E-41 | 1.05E-38 | 83.6967 |
| GBP5 | 1.746371 | 9.329632 | 14.84353 | 7.15E-41 | 5.26E-38 | 82.06708 |
| GIMAP1 | 1.25913 | 8.118576 | 14.83838 | 7.53E-41 | 5.31E-38 | 82.01608 |
| GPR171 | 1.755769 | 8.482403 | 14.65508 | 4.71E-40 | 3.19E-37 | 80.20648 |
| GGTA1P | 1.375425 | 8.956302 | 14.4349 | 4.21E-39 | 2.75E-36 | 78.04335 |
| TNFSF13B | 1.382084 | 11.36066 | 14.2851 | 1.85E-38 | 1.17E-35 | 76.57833 |
| IPCEF1 | 1.544917 | 7.799689 | 14.20807 | 3.97E-38 | 2.34E-35 | 75.82718 |
| CCL5 | 1.496259 | 11.71746 | 14.20791 | 3.97E-38 | 2.34E-35 | 75.82561 |
| IL15 | 1.144496 | 8.026969 | 14.01005 | 2.78E-37 | 1.58E-34 | 73.90339 |
| SLC27A2 | 1.339411 | 7.289059 | 13.86326 | 1.17E-36 | 6.46E-34 | 72.48406 |
| TC2N | 1.620979 | 6.102289 | 13.82739 | 1.66E-36 | 8.89E-34 | 72.13809 |
| GBP1 | 1.358903 | 10.32194 | 13.78404 | 2.54E-36 | 1.32E-33 | 71.72052 |
| GBP4 | 1.449706 | 8.326569 | 13.76545 | 3.04E-36 | 1.53E-33 | 71.54165 |
| CXCL10 | 1.752849 | 12.09999 | 13.66706 | 7.93E-36 | 3.78E-33 | 70.59632 |
| GZMB | 1.774943 | 11.1409 | 13.45772 | 6.02E-35 | 2.78E-32 | 68.59424 |
| CXCL11 | 1.853781 | 9.101009 | 13.45572 | 6.14E-35 | 2.78E-32 | 68.57516 |
| CPVL | 1.354588 | 9.868476 | 13.44932 | 6.53E-35 | 2.88E-32 | 68.51418 |
| **id** | **logFC** | **AveExpr** | **t** | **P.Value** | **adj.P.Val** | **B** |
| SLAMF7 | 1.750565 | 9.232389 | 13.40802 | 9.73E-35 | 4.08E-32 | 68.12091 |
| ANKRD22 | 1.636146 | 9.382509 | 13.40159 | 1.03E-34 | 4.24E-32 | 68.05969 |
| GPR155 | 1.054793 | 6.910948 | 13.38036 | 1.27E-34 | 5.09E-32 | 67.85777 |
| CLEC2B | 1.701253 | 8.586322 | 13.22024 | 5.91E-34 | 2.27E-31 | 66.33905 |
| BCL11B | 1.202672 | 7.440356 | 13.10657 | 1.75E-33 | 6.58E-31 | 65.26576 |
| LCP2 | 1.002349 | 7.839957 | 13.03321 | 3.53E-33 | 1.30E-30 | 64.57527 |
| AIF1 | 1.057333 | 10.01736 | 13.031 | 3.60E-33 | 1.30E-30 | 64.55448 |
| DDX60 | 1.236635 | 9.164565 | 12.9722 | 6.31E-33 | 2.22E-30 | 64.00234 |
| GBP2 | 1.094287 | 10.16106 | 12.89289 | 1.34E-32 | 4.63E-30 | 63.25932 |
| CCR5 | 1.231425 | 10.35117 | 12.74093 | 5.63E-32 | 1.87E-29 | 61.84164 |
| TRBC1 | 1.334184 | 11.77415 | 12.64206 | 1.43E-31 | 4.58E-29 | 60.92344 |
| IGSF6 | 1.339594 | 9.112613 | 12.59436 | 2.23E-31 | 7.03E-29 | 60.48173 |
| XCL1 | 1.320592 | 7.011635 | 12.49306 | 5.76E-31 | 1.75E-28 | 59.54617 |
| TSPAN5 | 1.070205 | 7.006283 | 12.44064 | 9.40E-31 | 2.81E-28 | 59.06345 |
| JAKMIP1 | 1.459287 | 7.033615 | 12.42239 | 1.11E-30 | 3.27E-28 | 58.89559 |
| IDO1 | 1.439917 | 9.845587 | 12.34548 | 2.28E-30 | 6.59E-28 | 58.18976 |
| FASLG | 1.032485 | 7.523903 | 12.32688 | 2.71E-30 | 7.70E-28 | 58.01929 |
| VAMP5 | 1.132578 | 7.00546 | 12.26844 | 4.65E-30 | 1.30E-27 | 57.4848 |
| GBP3 | 1.196679 | 9.902768 | 12.18098 | 1.04E-29 | 2.88E-27 | 56.68717 |
| CD96 | 1.294172 | 7.670555 | 12.15128 | 1.37E-29 | 3.67E-27 | 56.41687 |
| CD247 | 1.209496 | 9.765014 | 12.0973 | 2.26E-29 | 5.94E-27 | 55.92666 |
| ICOS | 2.044213 | 7.412886 | 12.08286 | 2.58E-29 | 6.59E-27 | 55.79571 |
| PRKCQ | 1.042233 | 7.953663 | 12.0734 | 2.81E-29 | 7.09E-27 | 55.70994 |
| **id** | **logFC** | **AveExpr** | **t** | **P.Value** | **adj.P.Val** | **B** |
| CXCL9 | 1.597949 | 13.59907 | 12.03897 | 3.86E-29 | 9.58E-27 | 55.3981 |
| DTHD1 | 1.909234 | 4.708095 | 11.99746 | 5.65E-29 | 1.38E-26 | 55.02274 |
| CCL4 | 1.146113 | 10.82941 | 11.97061 | 7.22E-29 | 1.74E-26 | 54.78037 |
| FCGR1B | 1.739351 | 9.225228 | 11.9275 | 1.07E-28 | 2.52E-26 | 54.39171 |
| TLR8 | 1.587627 | 7.929804 | 11.87195 | 1.78E-28 | 4.12E-26 | 53.89196 |
| TIGIT | 1.215817 | 9.54828 | 11.80191 | 3.36E-28 | 7.57E-26 | 53.2636 |
| CRY1 | 1.156362 | 8.534802 | 11.70336 | 8.20E-28 | 1.79E-25 | 52.3828 |
| LAG3 | 1.445849 | 9.327372 | 11.64859 | 1.35E-27 | 2.86E-25 | 51.89492 |
| TMEM71 | 1.818195 | 6.900383 | 11.6321 | 1.56E-27 | 3.28E-25 | 51.74832 |
| MAF | 1.029228 | 5.986893 | 11.45327 | 7.77E-27 | 1.56E-24 | 50.16527 |
| CD28 | 1.331703 | 6.136107 | 11.42355 | 1.01E-26 | 1.99E-24 | 49.90351 |
| GATA3 | 1.285379 | 6.908675 | 11.40863 | 1.16E-26 | 2.24E-24 | 49.77224 |
| RGL4 | 1.775169 | 5.382617 | 11.36995 | 1.63E-26 | 3.13E-24 | 49.43224 |
| ST8SIA1 | 1.379004 | 6.109914 | 11.36655 | 1.69E-26 | 3.20E-24 | 49.4024 |
| LILRB2 | 1.269151 | 8.762499 | 11.27919 | 3.66E-26 | 6.80E-24 | 48.63717 |
| SCML1 | 1.208406 | 7.162728 | 11.27008 | 3.97E-26 | 7.29E-24 | 48.55757 |
| PLA2G4A | 1.158084 | 6.848998 | 11.23694 | 5.32E-26 | 9.68E-24 | 48.2683 |
| PRF1 | 1.032936 | 10.28535 | 11.16878 | 9.72E-26 | 1.75E-23 | 47.67478 |
| TMEM155 | 1.403377 | 7.405156 | 11.09968 | 1.79E-25 | 3.15E-23 | 47.07513 |
| NKG7 | 1.368494 | 11.02064 | 11.04415 | 2.91E-25 | 4.98E-23 | 46.59475 |
| C1QB | 1.835869 | 11.56602 | 10.97234 | 5.45E-25 | 8.98E-23 | 45.97566 |
| PSTPIP2 | 1.30049 | 8.490965 | 10.84539 | 1.65E-24 | 2.66E-22 | 44.88682 |
| FGFBP2 | 1.549764 | 5.823623 | 10.80684 | 2.30E-24 | 3.65E-22 | 44.55761 |
| **id** | **logFC** | **AveExpr** | **t** | **P.Value** | **adj.P.Val** | **B** |
| KCNJ2 | 1.168243 | 8.270852 | 10.79874 | 2.46E-24 | 3.88E-22 | 44.48854 |
| ASCL2 | 1.022141 | 4.012346 | 10.7804 | 2.89E-24 | 4.51E-22 | 44.33228 |
| TTC16 | 1.466657 | 4.426063 | 10.7775 | 2.96E-24 | 4.58E-22 | 44.30759 |
| CCRL2 | 1.049175 | 8.174891 | 10.74546 | 3.91E-24 | 5.84E-22 | 44.03494 |
| C3AR1 | 1.092941 | 9.753192 | 10.69887 | 5.83E-24 | 8.50E-22 | 43.63937 |
| ITK | 1.262695 | 9.691843 | 10.66705 | 7.67E-24 | 1.10E-21 | 43.36974 |
| RGS18 | 1.36525 | 7.978316 | 10.61474 | 1.20E-23 | 1.69E-21 | 42.92767 |
| LAT | 1.126614 | 8.573288 | 10.61416 | 1.21E-23 | 1.69E-21 | 42.92274 |
| NAP1L2 | 1.210961 | 6.265048 | 10.61146 | 1.24E-23 | 1.72E-21 | 42.89995 |
| GZMM | 1.42347 | 7.635157 | 10.5201 | 2.70E-23 | 3.63E-21 | 42.13101 |
| ELOVL4 | 1.574918 | 5.192357 | 10.45319 | 4.77E-23 | 6.32E-21 | 41.57038 |
| CTSW | 1.639521 | 7.201466 | 10.40803 | 6.99E-23 | 9.13E-21 | 41.19318 |
| CD160 | 1.174757 | 6.447682 | 10.33216 | 1.33E-22 | 1.70E-20 | 40.56182 |
| CCL8 | 1.595983 | 9.125369 | 10.19193 | 4.31E-22 | 5.13E-20 | 39.40235 |
| ASCL1 | 1.22588 | 5.65784 | 10.01603 | 1.86E-21 | 2.01E-19 | 37.96193 |
| GPR174 | 1.006346 | 6.509152 | 9.976462 | 2.58E-21 | 2.76E-19 | 37.64009 |
| ARHGAP18 | 1.028723 | 8.654942 | 9.604874 | 5.35E-20 | 5.12E-18 | 34.65781 |
| MRC1 | 1.287244 | 9.075173 | 9.559744 | 7.69E-20 | 7.29E-18 | 34.30064 |
| PHACTR2 | 1.066184 | 8.300962 | 9.499341 | 1.25E-19 | 1.15E-17 | 33.82433 |
| MS4A4A | 1.202851 | 8.83111 | 9.343209 | 4.32E-19 | 3.79E-17 | 32.6025 |
| GBP1P1 | 1.275267 | 4.748016 | 9.338998 | 4.47E-19 | 3.90E-17 | 32.56974 |
| CD79A | -1.08118 | 12.29641 | -9.2675 | 7.86E-19 | 6.66E-17 | 32.01491 |
| GNLY | 1.427898 | 9.01987 | 9.178014 | 1.59E-18 | 1.30E-16 | 31.32464 |
| **id** | **logFC** | **AveExpr** | **t** | **P.Value** | **adj.P.Val** | **B** |
| VPS37B | 1.027511 | 7.686425 | 9.173564 | 1.64E-18 | 1.34E-16 | 31.29043 |
| RAB37 | 1.041842 | 7.644353 | 9.141319 | 2.11E-18 | 1.69E-16 | 31.04288 |
| CP | 1.294294 | 7.447803 | 9.115655 | 2.58E-18 | 2.04E-16 | 30.8463 |
| ABCD2 | 1.140198 | 5.388462 | 8.942757 | 9.84E-18 | 7.14E-16 | 29.53182 |
| B3GAT1 | 1.250662 | 4.913678 | 8.865471 | 1.78E-17 | 1.23E-15 | 28.94991 |
| P2RY14 | 1.177132 | 9.35052 | 8.824156 | 2.44E-17 | 1.65E-15 | 28.64028 |
| CD22 | -1.2043 | 9.131735 | -8.821 | 2.50E-17 | 1.68E-15 | 28.61664 |
| DDX11 | -1.02008 | 8.417912 | -8.79907 | 2.95E-17 | 1.97E-15 | 28.45281 |
| PTGER2 | 1.032017 | 7.184823 | 8.748779 | 4.32E-17 | 2.83E-15 | 28.07802 |
| S100B | 1.272128 | 4.42662 | 8.747629 | 4.36E-17 | 2.84E-15 | 28.06947 |
| PTPRN2 | 1.019346 | 6.35189 | 8.74661 | 4.40E-17 | 2.85E-15 | 28.06189 |
| DUSP4 | 1.062776 | 7.868506 | 8.73047 | 4.97E-17 | 3.20E-15 | 27.94196 |
| CD14 | 1.030075 | 11.51611 | 8.63477 | 1.02E-16 | 6.32E-15 | 27.23409 |
| CD40LG | 1.169868 | 5.812393 | 8.467755 | 3.55E-16 | 2.03E-14 | 26.01213 |
| CD33 | 1.188073 | 6.473311 | 8.465084 | 3.62E-16 | 2.07E-14 | 25.99272 |
| MS4A7 | 1.128262 | 8.47151 | 8.448771 | 4.08E-16 | 2.29E-14 | 25.87431 |
| RAB38 | 1.032667 | 6.767633 | 8.243884 | 1.83E-15 | 9.40E-14 | 24.40139 |
| IFIT1 | 1.314213 | 7.930463 | 8.156919 | 3.44E-15 | 1.70E-13 | 23.78424 |
| FCHO2 | 1.181 | 8.312541 | 8.155701 | 3.47E-15 | 1.71E-13 | 23.77562 |
| KLRC3 | 1.287531 | 5.606604 | 8.112086 | 4.75E-15 | 2.25E-13 | 23.46797 |
| PLAC8 | 1.355879 | 9.317652 | 7.995783 | 1.09E-14 | 4.81E-13 | 22.6536 |
| LGALS2 | 1.394334 | 8.524104 | 7.985982 | 1.17E-14 | 5.14E-13 | 22.58537 |
| ALDH1A1 | 1.056215 | 8.66716 | 7.91886 | 1.88E-14 | 8.00E-13 | 22.11982 |
| **id** | **logFC** | **AveExpr** | **t** | **P.Value** | **adj.P.Val** | **B** |
| CHRDL1 | 1.085752 | 7.7159 | 7.851801 | 3.02E-14 | 1.22E-12 | 21.65766 |
| KLRB1 | 1.148517 | 8.106355 | 7.750423 | 6.12E-14 | 2.30E-12 | 20.96465 |
| S100A9 | 1.314985 | 10.38399 | 7.562987 | 2.23E-13 | 7.41E-12 | 19.70154 |
| MALAT1 | 1.052321 | 10.72142 | 7.555836 | 2.34E-13 | 7.75E-12 | 19.65382 |
| MAL | 1.03499 | 8.514292 | 7.515883 | 3.07E-13 | 9.83E-12 | 19.38786 |
| IFI44L | 1.152132 | 8.573102 | 7.347124 | 9.56E-13 | 2.77E-11 | 18.2766 |
| IRX3 | 1.046094 | 6.536926 | 7.303951 | 1.28E-12 | 3.59E-11 | 17.99549 |
| S100A8 | 1.399311 | 8.586991 | 7.289541 | 1.40E-12 | 3.92E-11 | 17.90194 |
| BEX5 | 1.134634 | 7.198131 | 7.222923 | 2.18E-12 | 5.86E-11 | 17.47141 |
| PMCH | 1.104668 | 6.579483 | 7.200302 | 2.53E-12 | 6.69E-11 | 17.32592 |
| PDCD1LG2 | 1.054372 | 5.023997 | 7.174176 | 3.00E-12 | 7.77E-11 | 17.15835 |
| LIMD2 | -1.0293 | 8.98537 | -7.12501 | 4.15E-12 | 1.03E-10 | 16.8443 |
| KIF21A | 1.098927 | 6.658529 | 7.120845 | 4.26E-12 | 1.05E-10 | 16.81778 |
| VSIG4 | 1.424537 | 9.205103 | 7.03947 | 7.23E-12 | 1.69E-10 | 16.30203 |
| FCGR1A | 1.113034 | 4.251649 | 7.035232 | 7.43E-12 | 1.73E-10 | 16.27529 |
| LILRB5 | 1.068494 | 6.166267 | 7.031515 | 7.61E-12 | 1.76E-10 | 16.25186 |
| KCNA3 | 1.189958 | 6.482562 | 6.999075 | 9.38E-12 | 2.13E-10 | 16.04777 |
| CD163 | 1.161735 | 9.075234 | 6.93183 | 1.44E-11 | 3.10E-10 | 15.62711 |
| PRAM1 | 1.023876 | 4.883831 | 6.910246 | 1.66E-11 | 3.51E-10 | 15.49279 |
| ST6GALNAC1 | 1.018787 | 3.676652 | 6.897601 | 1.80E-11 | 3.74E-10 | 15.41425 |
| DTX1 | -1.10667 | 10.23553 | -6.8593 | 2.29E-11 | 4.62E-10 | 15.17708 |
| IL1R2 | 1.042652 | 7.044124 | 6.721979 | 5.43E-11 | 1.01E-09 | 14.33554 |
| NME8 | 1.375183 | 3.880248 | 6.570492 | 1.39E-10 | 2.37E-09 | 13.42336 |
| **id** | **logFC** | **AveExpr** | **t** | **P.Value** | **adj.P.Val** | **B** |
| SIGLEC1 | 1.024714 | 7.89567 | 6.553799 | 1.53E-10 | 2.60E-09 | 13.32388 |
| KLRC4 | 1.057766 | 4.960276 | 6.404462 | 3.80E-10 | 5.84E-09 | 12.44332 |
| DDX43 | 1.082422 | 4.72804 | 6.262874 | 8.82E-10 | 1.25E-08 | 11.62408 |
| BEX2 | 1.094502 | 8.867491 | 6.140777 | 1.80E-09 | 2.34E-08 | 10.92996 |
| NPY1R | 1.241119 | 5.417914 | 5.936868 | 5.80E-09 | 6.71E-08 | 9.79658 |
| MT1M | 1.333032 | 6.331279 | 5.438838 | 8.81E-08 | 7.72E-07 | 7.167178 |
| SCN3A | 1.027662 | 3.945643 | 5.063213 | 6.02E-07 | 4.26E-06 | 5.317836 |
| MARCO | 1.087038 | 7.101541 | 4.553657 | 6.79E-06 | 3.67E-05 | 2.998971 |
| HTR3A | -1.02938 | 8.084851 | -4.20682 | 3.13E-05 | 0.000142 | 1.548932 |

**Supplemental Table 3 Uni-Cox analysis**

| **Clinical characteristics** | **HR** | **HR.95L** | **HR.95H** | **P value** |
| --- | --- | --- | --- | --- |
| Gender | 0.93 | 0.65 | 1.32 | 0.668 |
| Age | 1.99 | 1.39 | 2.84 | <0.001 |
| ECOG performance status | 1.83 | 1.52 | 2.20 | <0.001 |
| Clinical stage | 1.50 | 1.26 | 1.79 | <0.001 |
| LDH ratio | 1.14 | 1.10 | 1.19 | <0.001 |
| Number of extranodal sites | 1.21 | 0.99 | 1.48 | 0.063 |
| CCL8 | 2.26 | 1.11 | 4.60 | 0.025 |

**Supplemental Table 4 Multi-Cox analysis**

| **Clinical characteristics** | **HR** | **HR.95L** | **HR.95H** | **P value** |
| --- | --- | --- | --- | --- |
| Age | 1.84 | 1.27 | 2.65 | 0.001 |
| ECOG performance status | 1.56 | 1.29 | 1.90 | <0.001 |
| Clinical stage | 1.31 | 1.09 | 1.57 | 0.004 |
| LDH ratio | 1.15 | 1.09 | 1.21 | <0.001 |
| CCL8 | 2.78 | 1.33 | 5.82 | 0.007 |

**Supplementary Table 5: Gene mutation related to CCL8 expression**

| **Gene** | **Number of mutations** | **Gene** | **Number of mutations** |
| --- | --- | --- | --- |
| KMT2D | 9 | IGHM | 4 |
| BTG2 | 6 | MPEG1 | 4 |
| IGHG2 | 6 | IGLV3-1 | 4 |
| KLHL6 | 6 | TTN | 4 |
| ZFHX4 | 6 | B2M | 4 |
| NFKBIE | 6 | IGHG1 | 4 |
| H1-4 | 6 | ASXL3 | 4 |
| CARD11 | 5 | IGLC3 | 4 |
| CREBBP | 5 | UACA | 4 |
| IRF4 | 5 | UBR4 | 4 |
| TMSB4X | 5 | DDX3X | 3 |
| CSMD3 | 5 | ADCY2 | 3 |
| SOCS1 | 5 | STAT6 | 3 |
| IGHE | 4 | PIM1 | 3 |
| LRRN3 | 4 | ARID1A | 3 |
| IRF8 | 4 | ADGRB3 | 3 |
| PCLO | 4 | COL3A1 | 3 |
| CSNK2A1 | 4 | SPTBN4 | 3 |
| GABRG3 | 4 | CD79B | 3 |
| H1-3 | 4 | P2RY8 | 3 |
| ATP10B | 3 | BCL7A | 2 |
| Gene | Number of mutations | Gene | Number of mutations |
| LRP2 | 3 | IGKV1D-13 | 2 |
| H1-2 | 3 | LIMD2 | 2 |
| IGKC | 3 | IGKV1-5 | 2 |
| ZNF208 | 3 | CD70 | 2 |
| MUC16 | 3 | SCN1A | 2 |
| CXCR4 | 3 | ZSCAN1 | 2 |
| DNAH9 | 3 | MUC5B | 2 |
| IGHV2-70 | 3 | CACNA1H | 2 |
| SCN7A | 3 | CIITA | 2 |
| DUSP2 | 3 | PLD2 | 2 |
| MPDZ | 3 | DSEL | 2 |
| STAG2 | 3 | SMC2 | 2 |
| IGHV1-69 | 3 | HTT | 2 |
| PLCE1 | 3 | DENND4C | 2 |
| TNFRSF14 | 3 | BTK | 2 |
| CSK | 3 | IGHA1 | 2 |
| ARHGAP21 | 3 | HPSE2 | 2 |
| TMEM30A | 3 | AGTR2 | 2 |
| IGHG4 | 3 | AASS | 2 |
| CCDC88B | 3 | IGHG3 | 2 |
| KLF2 | 3 | FLG | 2 |
| PCDH15 | 3 | IGLV4-69 | 2 |
| SCN9A | 3 | RAF1 | 2 |
| FLNC | 3 | ROBO1 | 2 |
| ATR | 3 | CDK5R1 | 2 |
| MAST4 | 3 | MAGEL2 | 2 |
| DNAH5 | 3 | PCSK5 | 2 |
| CHRM3 | 3 | NFKBIA | 2 |
| SYNE1 | 3 | JARID2 | 2 |
| BTG1 | 3 | NOTCH2 | 2 |
| CNTNAP5 | 3 | PPFIA2 | 2 |
| IGLL5 | 3 | CDH11 | 2 |
| MTMR1 | 3 | TRPM6 | 2 |
| KLHL14 | 3 | SLC6A15 | 2 |
| TNFAIP3 | 3 | H2AC15 | 2 |
| MPP7 | 3 | DGAT2L6 | 2 |
| CTNND2 | 2 | MUC6 | 2 |
| CASR | 2 | AMOT | 2 |
| FOXC1 | 2 | STXBP3 | 2 |
| EGR1 | 2 | COL21A1 | 2 |
| AGBL1 | 2 | GRIN2A | 2 |
| DGKI | 2 | EXOC4 | 2 |
| Gene | Number of mutations | Gene | Number of mutations |
| EPHB2 | 2 | ERBIN | 2 |
| PCDHA5 | 2 | BCL9L | 2 |
| MSL2 | 2 | GRID1 | 2 |
| DTX3L | 2 | CD58 | 2 |
| NOX4 | 2 | DIAPH3 | 2 |
| USP9X | 2 | WNK1 | 2 |
| ACTB | 2 | MAGI1 | 2 |
| CDH26 | 2 | LRFN5 | 2 |
| CEP85 | 2 | LRP1B | 2 |
| PCDHA12 | 2 | FAM120A | 2 |
| MSN | 2 | ENOX1 | 2 |
| LRRTM1 | 2 | H4-16 | 2 |
| PCDH11X | 2 | CACNA1E | 2 |
| UNC13C | 2 | STAP2 | 2 |
| HEPH | 2 | SEMA3C | 2 |
| ATP6V0A1 | 2 | ALDH5A1 | 2 |
| CDH20 | 2 | CHD5 | 2 |
| TRIO | 2 | ZC3H7A | 2 |
| CHD3 | 2 | H2BC12 | 2 |
| KIF13A | 2 | IGLV3-25 | 2 |
| ATP13A5 | 2 | XIRP2 | 2 |
| CPAMD8 | 2 | ZNF708 | 2 |
| ANKHD1-EIF4EBP3 | 2 | ABCB5 | 2 |
| CNTN1 | 2 | KIF2B | 2 |
| DSC1 | 2 | CNTRL | 2 |
| SGK1 | 2 | CMYA5 | 2 |
| PRKN | 2 | FMNL2 | 2 |
| ZNF804B | 2 | PRRC2B | 2 |
| MEF2B | 2 | MED12 | 2 |
| ZBTB40 | 2 | PRAMEF20 | 2 |
| IGKV4-1 | 2 | ADCK1 | 2 |
| INPP5F | 2 | JUNB | 2 |
| H4C6 | 2 | IGHV4-34 | 2 |
| HERC4 | 2 | SI | 2 |
| NAV3 | 2 | CHRNA2 | 2 |
| IGHA2 | 2 | MIPEP | 2 |
| TOGARAM1 | 2 | UBR5 | 2 |
| CAVIN2 | 2 | USH2A | 2 |
| DSG3 | 2 | SLC32A1 | 2 |
| SLC37A3 | 2 | INTS3 | 2 |
| ALMS1 | 2 | MYO9A | 2 |
| Gene | Number of mutations | Gene | Number of mutations |
| MSLN | 2 | HERC1 | 2 |
| MME | 2 | SLC35F1 | 2 |
| DCAF8 | 2 | FAT4 | 2 |
| MAZ | 2 | FAM135B | 2 |
| KLHL1 | 2 | TENM4 | 2 |
| HLA-DQB1 | 2 | ANKRD11 | 2 |
| ITPKB | 2 | FAT1 | 2 |
| NEK1 | 2 | PPP1R3A | 2 |
| EPS8L2 | 2 | IKZF3 | 2 |
| PARD3 | 2 | ZNF217 | 2 |
| SCN3A | 2 | PCDH7 | 2 |
| HMCN1 | 2 | ZBTB20 | 2 |
| IGLC2 | 2 | DSG1 | 2 |
| BRD2 | 2 | CSMD1 | 2 |
| ANO5 | 2 | NCAM2 | 2 |
| AVIL | 2 | SALL1 | 2 |
| SMARCA4 | 2 | HKDC1 | 2 |
| BRCA1 | 2 | PAX5 | 2 |
| LAMA1 | 2 | ZAN | 2 |
| RIMS1 | 2 | MCM3AP | 2 |
| EBF1 | 2 | TRANK1 | 2 |
| LAMA5 | 2 | MYO9B | 2 |
| DOCK5 | 2 | CDAN1 | 2 |
| SRP72 | 2 | NEGR1 | 2 |
| STYXL2 | 2 | SLC16A7 | 2 |
| CNTNAP4 | 2 | SCNN1G | 2 |
| CTRC | 2 | FKBP9 | 2 |
| PHOX2B | 2 | TMEM201 | 2 |
| PLCB1 | 2 | FLNA | 2 |
| USP43 | 2 | CNTLN | 2 |
| OSBPL10 | 2 | GNA13 | 2 |
| LRRIQ1 | 2 | PCNX3 | 2 |
| ZC3H12A | 2 | H2AC17 | 2 |
| OR2L2 | 2 | RGL3 | 2 |
| PHF12 | 2 | POLQ | 2 |
| NXF1 | 2 | TRPM3 | 2 |
| BRAF | 2 | ARHGAP6 | 2 |
| FBP2 | 2 | MDGA2 | 2 |
| KCNJ8 | 2 | SPEN | 2 |
| TLN2 | 2 | ZC3H4 | 2 |
| THSD7A | 2 | JMJD1C | 2 |
| IGKV2-30 | 2 | TLL2 | 2 |
| Gene | Number of mutations | Gene | Number of mutations |
| MYH14 | 2 | CCDC39 | 2 |
| RIMS2 | 2 | PLXNB2 | 2 |
| SWAP70 | 2 | ATG4C | 2 |
| H2BC4 | 2 | DGKH | 1 |
| PGBD4 | 2 | CASZ1 | 1 |
| DSCC1 | 2 | FNDC7 | 1 |
| HLA-A | 2 | ZNF615 | 1 |
| KRAS | 2 | OR52R1 | 1 |
| PRMT9 | 2 | PTPRD | 1 |
| H2AC6 | 2 | OR8B2 | 1 |
| NETO1 | 2 | ATRIP | 1 |
| PKHD1L1 | 1 | UGT2B7 | 1 |
| PLEKHM3 | 1 | GCM2 | 1 |
| PLD6 | 1 | LIPM | 1 |
| ESRP1 | 1 | TDP1 | 1 |
| CCND2 | 1 | POLR2B | 1 |
| C5 | 1 | ZNF91 | 1 |
| OR5M1 | 1 | AMY2B | 1 |
| AHCYL1 | 1 | LPCAT2 | 1 |
| ATL3 | 1 | FBXW12 | 1 |
| RALGAPA2 | 1 | MGAM | 1 |
| RPL27A | 1 | IRF1 | 1 |
| ZNF461 | 1 | TOGARAM2 | 1 |
| MYL6 | 1 | TAC3 | 1 |
| CAMTA1 | 1 | UCN | 1 |
| AASDHPPT | 1 | EHD1 | 1 |
| ABLIM1 | 1 | SH3RF2 | 1 |
| TNFAIP8L2 | 1 | MYO6 | 1 |
| FBH1 | 1 | PHC1 | 1 |
| LIMK1 | 1 | DRG2 | 1 |
| CYB5R4 | 1 | RAB36 | 1 |
| MTSS1 | 1 | SMARCB1 | 1 |
| SLC7A5 | 1 | CTNNA3 | 1 |
| MYOM1 | 1 | GGNBP2 | 1 |
| HYDIN | 1 | WRAP53 | 1 |
| SMARCD3 | 1 | FLT1 | 1 |
| MINAR1 | 1 | COL9A1 | 1 |
| UBB | 1 | H2BC5 | 1 |
| SLC4A1 | 1 | ABCB9 | 1 |
| CPZ | 1 | GABRB1 | 1 |
| INO80D | 1 | EBF3 | 1 |
| ITPR3 | 1 | OPCML | 1 |
| Gene | Number of mutations | Gene | Number of mutations |
| TERB2 | 1 | H2BC11 | 1 |
| ARL6IP4 | 1 | AKR1C1 | 1 |
| LAMB1 | 1 | FAU | 1 |
| ZMYM3 | 1 | STK10 | 1 |
| PCDHA4 | 1 | ITGA10 | 1 |
| GRID2 | 1 | CLRN3 | 1 |
| CALY | 1 | NRXN1 | 1 |
| PGBD1 | 1 | DSCAML1 | 1 |
| KLHL17 | 1 | RAD51AP1 | 1 |
| VMA21 | 1 | TREX2 | 1 |
| SPTA1 | 1 | MCF2L | 1 |
| RAPH1 | 1 | NR2F2 | 1 |
| KDM2A | 1 | SPATA18 | 1 |
| NF1 | 1 | MAP7D2 | 1 |
| EPC2 | 1 | IBA57 | 1 |
| MCM7 | 1 | CAMK2G | 1 |
| OR1L3 | 1 | DNAH10 | 1 |
| CHEK2 | 1 | AIDA | 1 |
| BMS1 | 1 | NEFL | 1 |
| EPHA4 | 1 | EN1 | 1 |
| IGLV3-9 | 1 | OGG1 | 1 |
| PPP3R2 | 1 | TJP1 | 1 |
| SETX | 1 | SEMA3E | 1 |
| TP53 | 1 | COL14A1 | 1 |
| KCNK18 | 1 | CADM1 | 1 |
| CSN3 | 1 | PSMB5 | 1 |
| CIC | 1 | TLR2 | 1 |
| PROKR1 | 1 | ATP5ME | 1 |
| CELSR3 | 1 | THRB | 1 |
| COL5A3 | 1 | CDC37L1 | 1 |
| FKBP15 | 1 | CNDP1 | 1 |
| ZNF367 | 1 | DYNC2I2 | 1 |
| FPR3 | 1 | PKN3 | 1 |
| MAGI2 | 1 | CCND3 | 1 |
| FANCG | 1 | OR14J1 | 1 |
| ZNF354C | 1 | NSDHL | 1 |
| BIRC7 | 1 | MESD | 1 |
| BCKDHB | 1 | IL5RA | 1 |
| ALDH9A1 | 1 | TLN1 | 1 |
| RGS14 | 1 | EXD1 | 1 |
| RNPS1 | 1 | ADCY3 | 1 |
| CHM | 1 | CISH | 1 |
| Gene | Number of mutations | Gene | Number of mutations |
| USP25 | 1 | CYBB | 1 |
| HMGCR | 1 | PINK1 | 1 |
| PIK3C2A | 1 | PLEKHO2 | 1 |
| KIAA1549L | 1 | SLC25A13 | 1 |
| REV1 | 1 | MINDY2 | 1 |
| ADAD2 | 1 | LIMS1 | 1 |
| CLTB | 1 | TAF1 | 1 |
| OR2AK2 | 1 | LRRTM4 | 1 |
| ITPR2 | 1 | ILK | 1 |
| SNX18 | 1 | MARK1 | 1 |
| CPEB4 | 1 | MID2 | 1 |
| SLC25A52 | 1 | LCA5L | 1 |
| BIVM-ERCC5 | 1 | UBQLN2 | 1 |
| BARHL2 | 1 | SLC45A2 | 1 |
| BHLHA15 | 1 | NUP214 | 1 |
| GAD1 | 1 | FOXD4 | 1 |
| CAPZA3 | 1 | RPL8 | 1 |
| PPP1R16B | 1 | KLF15 | 1 |
| GP2 | 1 | MASTL | 1 |
| IGKV3-20 | 1 | MROH2B | 1 |
| WRAP73 | 1 | DAZAP1 | 1 |
| KIFC3 | 1 | TMEM108 | 1 |
| ITLN1 | 1 | GABRA5 | 1 |
| NFYB | 1 | VCAM1 | 1 |
| AP002748.5 | 1 | PGAP6 | 1 |
| ABCB8 | 1 | TNFRSF13C | 1 |
| EPSTI1 | 1 | NPAS1 | 1 |
| MAGI3 | 1 | STOML2 | 1 |
| BCL11B | 1 | PDE1A | 1 |
| ESYT3 | 1 | LRIT1 | 1 |
| PTEN | 1 | OR56A3 | 1 |
| MYC | 1 | FRMPD3 | 1 |
| IDH3B | 1 | RPS6KA5 | 1 |
| CD83 | 1 | SEMA5A | 1 |
| LRRC39 | 1 | MS4A8 | 1 |
| PLCG2 | 1 | CADPS2 | 1 |
| GCSAM | 1 | DOK5 | 1 |
| FOLH1 | 1 | PLCL2 | 1 |
| IFRD2 | 1 | ANKRD50 | 1 |
| PCDHA11 | 1 | NYAP2 | 1 |
| FAM186B | 1 | SYNGR2 | 1 |
| ZC3HAV1 | 1 | LARP1 | 1 |
| Gene | Number of mutations | Gene | Number of mutations |
| IGHV6-1 | 1 | PALD1 | 1 |
| HSPA6 | 1 | PSD4 | 1 |
| CCNB3 | 1 | DUOX1 | 1 |
| ARHGEF5 | 1 | MUSK | 1 |
| UBA6 | 1 | GALNT1 | 1 |
| FGF16 | 1 | EEF2K | 1 |
| KCNMB3 | 1 | GPAT2 | 1 |
| PPP2R2C | 1 | SLC27A5 | 1 |
| FOXA2 | 1 | AP3M1 | 1 |
| SON | 1 | TAF5L | 1 |
| ALPG | 1 | SPTBN5 | 1 |
| SNRNP35 | 1 | CTTNBP2 | 1 |
| PLEKHG6 | 1 | SLC5A9 | 1 |
| BRWD3 | 1 | RPL4 | 1 |
| TMEM135 | 1 | TELO2 | 1 |
| BMP5 | 1 | GRIN3A | 1 |
| CFHR5 | 1 | IGSF22 | 1 |
| NOL6 | 1 | KIF1A | 1 |
| ALX3 | 1 | TRDN | 1 |
| CD74 | 1 | MAP2K3 | 1 |
| SLC44A3 | 1 | ZNF846 | 1 |
| PTX3 | 1 | TTF1 | 1 |
| EPS8L1 | 1 | CNOT4 | 1 |
| AADACL3 | 1 | SNTB1 | 1 |
| LIPN | 1 | IMPG1 | 1 |
| BMP7 | 1 | ARHGAP28 | 1 |
| IRS4 | 1 | BRAP | 1 |
| COPRS | 1 | SGIP1 | 1 |
| FIP1L1 | 1 | LRPAP1 | 1 |
| ZNF569 | 1 | MSX1 | 1 |
| EPPK1 | 1 | NAPSA | 1 |
| DLGAP3 | 1 | SRPRB | 1 |
| GPC6 | 1 | DCHS1 | 1 |
| AXL | 1 | ZNF676 | 1 |
| ADGRG1 | 1 | FEM1B | 1 |
| F2R | 1 | TDRD5 | 1 |
| ZNF578 | 1 | PLK1 | 1 |
| UPF1 | 1 | PPP1CA | 1 |
| PCDH18 | 1 | ABHD2 | 1 |
| HARBI1 | 1 | CEBPZ | 1 |
| FANCL | 1 | MTNR1B | 1 |
| MTMR10 | 1 | TENM1 | 1 |
| Gene | Number of mutations | Gene | Number of mutations |
| CCSER1 | 1 | YIPF7 | 1 |
| CDH15 | 1 | EFNB1 | 1 |
| FAM178B | 1 | IGKV1-8 | 1 |
| WDR13 | 1 | GRM7 | 1 |
| ASCC3 | 1 | SH3TC1 | 1 |
| RTN2 | 1 | MRPL58 | 1 |
| MICAL1 | 1 | HIF3A | 1 |
| SSBP2 | 1 | NPEPPS | 1 |
| TPX2 | 1 | EIF4G2 | 1 |
| ZNF256 | 1 | BYSL | 1 |
| CHD2 | 1 | ABCA1 | 1 |
| PHKA1 | 1 | DGKD | 1 |
| FGFR4 | 1 | CRACDL | 1 |
| LCT | 1 | SHROOM3 | 1 |
| RAB6B | 1 | LRRFIP1 | 1 |
| LRRC7 | 1 | AOC1 | 1 |
| ERG | 1 | PGD | 1 |
| TSC22D2 | 1 | HOOK1 | 1 |
| DEFB114 | 1 | MAGEB6 | 1 |
| LNPEP | 1 | FRG2C | 1 |
| PTPRB | 1 | HNF4G | 1 |
| MOCS3 | 1 | EIF3L | 1 |
| NRK | 1 | UBN1 | 1 |
| ACOX3 | 1 | CYP46A1 | 1 |
| SLC20A1 | 1 | SLC22A23 | 1 |
| OXCT1 | 1 | BRINP2 | 1 |
| PRR5-ARHGAP8 | 1 | CASD1 | 1 |
| SEMA7A | 1 | XRCC5 | 1 |
| ZNF274 | 1 | NEB | 1 |
| RBM10 | 1 | FAM104A | 1 |
| NLRP9 | 1 | TMC5 | 1 |
| MGA | 1 | ITPRID1 | 1 |
| C1orf112 | 1 | MFSD14B | 1 |
| POLR2E | 1 | GPR171 | 1 |
| PLEKHS1 | 1 | NASP | 1 |
| TRMT13 | 1 | ACSL3 | 1 |
| CYSLTR1 | 1 | CSNK1A1L | 1 |
| PCDHGA12 | 1 | ZNF616 | 1 |
| VIT | 1 | H2AC21 | 1 |
| ATP2B4 | 1 | CILP2 | 1 |
| PROX1 | 1 | MYO1E | 1 |
| Gene | Number of mutations | Gene | Number of mutations |
| TRPV3 | 1 | SSBP3 | 1 |
| MMRN1 | 1 | PKD2 | 1 |
| CCDC171 | 1 | RALGDS | 1 |
| RASSF6 | 1 | DLX4 | 1 |
| DNAJB8 | 1 | PTPRC | 1 |
| ASPH | 1 | RNF19A | 1 |
| GRHPR | 1 | ZNF324 | 1 |
| ZNF385D | 1 | PEG3 | 1 |
| TFAP2B | 1 | CABCOCO1 | 1 |
| MFSD3 | 1 | AR | 1 |
| BCL3 | 1 | SCYL3 | 1 |
| SRP54 | 1 | AARS2 | 1 |
| ZNF608 | 1 | THEMIS | 1 |
| SLC26A7 | 1 | TSHR | 1 |
| RGS9 | 1 | KIAA1328 | 1 |
| ERCC6L2 | 1 | OR7G1 | 1 |
| ANKRD13A | 1 | RITA1 | 1 |
| COLEC12 | 1 | SIPA1L1 | 1 |
| HOXA11 | 1 | CDHR3 | 1 |
| KRT12 | 1 | SYT1 | 1 |
| SMG5 | 1 | LSM8 | 1 |
| C1QTNF2 | 1 | KRTAP5-2 | 1 |
| RAB9A | 1 | PTPN21 | 1 |
| PHF14 | 1 | ENOSF1 | 1 |
| VWA3A | 1 | ZFPM2 | 1 |
| SMARCA2 | 1 | KLHL32 | 1 |
| CCR2 | 1 | CSNK1E | 1 |
| VWDE | 1 | PRKG1 | 1 |
| CDH7 | 1 | KDR | 1 |
| SREBF2 | 1 | AL592490.1 | 1 |
| PTPRM | 1 | PTPN2 | 1 |
| ANGPTL2 | 1 | CNRIP1 | 1 |
| SLC8A1 | 1 | ZNF236 | 1 |
| PWWP3A | 1 | QTRT1 | 1 |
| POLR3F | 1 | PABPC3 | 1 |
| LINGO4 | 1 | EPHB1 | 1 |
| RPL13 | 1 | KCTD20 | 1 |
| MYO1A | 1 | RFX7 | 1 |
| NFATC2IP | 1 | CEMIP2 | 1 |
| TRAK1 | 1 | GRINA | 1 |
| DALRD3 | 1 | H2AC11 | 1 |
| IGLV5-37 | 1 | MKRN2 | 1 |
| Gene | Number of mutations | Gene | Number of mutations |
| XPO1 | 1 | TDRD15 | 1 |
| MAP4K1 | 1 | TBX5 | 1 |
| CFAP94 | 1 | CACNA1D | 1 |
| MET | 1 | DGKK | 1 |
| MAST1 | 1 | NPAT | 1 |
| ERC1 | 1 | ADAMTS20 | 1 |
| TRIM6-TRIM34 | 1 | FAM172A | 1 |
| TSHZ1 | 1 | DMP1 | 1 |
| PIK3CA | 1 | SETMAR | 1 |
| KCNN1 | 1 | MTTP | 1 |
| TMEM74 | 1 | LRPPRC | 1 |
| MBD3L1 | 1 | ZNF347 | 1 |
| WDR72 | 1 | BTBD9 | 1 |
| SYT17 | 1 | OR6S1 | 1 |
| SLC41A1 | 1 | FRAS1 | 1 |
| OR10A4 | 1 | KTN1 | 1 |
| CAPN12 | 1 | C1QBP | 1 |
| CETN3 | 1 | PBRM1 | 1 |
| MSI2 | 1 | ACADM | 1 |
| GPRIN2 | 1 | KIF4B | 1 |
| DOCK4 | 1 | CNPY4 | 1 |
| HECW1 | 1 | MAP3K10 | 1 |
| CHRNA6 | 1 | CEP126 | 1 |
| EEF2 | 1 | TRIM39 | 1 |
| RTN3 | 1 | SLC19A3 | 1 |
| PCF11 | 1 | PIWIL1 | 1 |
| SLC12A6 | 1 | ATF4 | 1 |
| DCT | 1 | RUNX1T1 | 1 |
| CALHM5 | 1 | XPO7 | 1 |
| KLHL18 | 1 | SLC6A14 | 1 |
| OR6B3 | 1 | PLIN3 | 1 |
| SPINK5 | 1 | RHOA | 1 |
| SLC45A1 | 1 | DUSP16 | 1 |
| LRCH1 | 1 | ARFGEF3 | 1 |
| OR8H1 | 1 | COL22A1 | 1 |
| VWA3B | 1 | PIK3CG | 1 |
| TBC1D23 | 1 | EPHA6 | 1 |
| PKHD1 | 1 | SRGAP1 | 1 |
| TMC1 | 1 | LRP3 | 1 |
| SMCR8 | 1 | ARMCX5 | 1 |
| SMC1B | 1 | MAGEB16 | 1 |
| Gene | Number of mutations | Gene | Number of mutations |
| ZNF106 | 1 | ZSCAN20 | 1 |
| TET2 | 1 | PIPOX | 1 |
| SNX27 | 1 | MAD2L1BP | 1 |
| VEPH1 | 1 | STOX2 | 1 |
| MAGEA4 | 1 | OGFR | 1 |
| TPR | 1 | TGFBR3 | 1 |
| PLXDC2 | 1 | CCDC81 | 1 |
| HTATSF1 | 1 | SPINDOC | 1 |
| ZFC3H1 | 1 | SPIN3 | 1 |
| SLC9B2 | 1 | AMELX | 1 |
| B3GNT8 | 1 | MAP7D3 | 1 |
| TAAR2 | 1 | FANCD2 | 1 |
| PCDHAC1 | 1 | HIPK3 | 1 |
| ASB17 | 1 | RGS6 | 1 |
| KLRK1 | 1 | ZDHHC17 | 1 |
| ZNF234 | 1 | DENND5B | 1 |
| CAD | 1 | TSKU | 1 |
| YES1 | 1 | FLVCR1 | 1 |
| ALG10 | 1 | CPN1 | 1 |
| NFIB | 1 | UNK | 1 |
| ABCA2 | 1 | ADARB2 | 1 |
| BCL6 | 1 | SFXN1 | 1 |
| ZDHHC6 | 1 | SDHC | 1 |
| NPAS3 | 1 | SH3RF3 | 1 |
| AMN1 | 1 | ZNF804A | 1 |
| CDK13 | 1 | COL16A1 | 1 |
| BMP4 | 1 | CTNNA2 | 1 |
| CBARP | 1 | SEC16A | 1 |
| ZC2HC1A | 1 | NUBPL | 1 |
| CTPS2 | 1 | IGLV4-3 | 1 |
| GOLGA3 | 1 | KAT2B | 1 |
| ZSCAN5B | 1 | TBC1D9 | 1 |
| MFHAS1 | 1 | DPP8 | 1 |
| TRIM24 | 1 | TAF1L | 1 |
| XRCC2 | 1 | PLEKHA6 | 1 |
| HOATZ | 1 | SLC47A1 | 1 |
| TASOR2 | 1 | OR51M1 | 1 |
| RAB40B | 1 | MED22 | 1 |
| DCHS2 | 1 | CNTN5 | 1 |
| TLE3 | 1 | BPIFB4 | 1 |
| ADGRV1 | 1 | DSCAM | 1 |
| ATP6V0A4 | 1 | UHRF1BP1 | 1 |
| Gene | Number of mutations | Gene | Number of mutations |
| PCDHGA11 | 1 | MEPCE | 1 |
| AKR1C4 | 1 | WDR53 | 1 |
| TOX | 1 | NAAA | 1 |
| CHD9 | 1 | OR52L1 | 1 |
| C18orf54 | 1 | PMPCB | 1 |
| PALB2 | 1 | GPR55 | 1 |
| PEX2 | 1 | BCL10 | 1 |
| XKR3 | 1 | DENR | 1 |
| PDZD4 | 1 | NYAP1 | 1 |
| COL5A2 | 1 | C6orf62 | 1 |
| IGLV3-16 | 1 | KCNE5 | 1 |
| SVIL | 1 | GRIN2B | 1 |
| ERBB4 | 1 | MNX1 | 1 |
| PLCL1 | 1 | COL23A1 | 1 |
| CCR3 | 1 | ESPL1 | 1 |
| PCOLCE | 1 | RAB29 | 1 |
| PANX2 | 1 | AHNAK | 1 |
| FOXD4L1 | 1 | INO80 | 1 |
| BCL2 | 1 | CFAP52 | 1 |
| CBWD6 | 1 | ECPAS | 1 |
| KRT73 | 1 | AGTR1 | 1 |
| GRM1 | 1 | SHANK1 | 1 |
| LRIT2 | 1 | COL6A1 | 1 |
| ABCA3 | 1 | PTPN14 | 1 |
| PLXNA4 | 1 | ATP6V1H | 1 |
| CDK12 | 1 | JMJD7-PLA2G4B | 1 |
| OR2L8 | 1 | ZNF501 | 1 |
| MCF2 | 1 | PLCG1 | 1 |
| VN1R1 | 1 | TACC2 | 1 |
| HSPA4 | 1 | LY9 | 1 |
| FLG2 | 1 | ANKFY1 | 1 |
| MYH1 | 1 | RALGAPA1 | 1 |
| RLF | 1 | NAGLU | 1 |
| PARD3B | 1 | OPTN | 1 |
| ZNF141 | 1 | ALOXE3 | 1 |
| CTSH | 1 | ARSF | 1 |
| IGHV4-59 | 1 | HMMR | 1 |
| SPAG17 | 1 | ADRA2B | 1 |
| UBTF | 1 | ARID5B | 1 |
| FBXO38 | 1 | DIRAS3 | 1 |
| FBXL20 | 1 | WFDC11 | 1 |
| Gene | Number of mutations | Gene | Number of mutations |
| PDE1B | 1 | LTB | 1 |
| SLC2A14 | 1 | ANGPTL7 | 1 |
| ANLN | 1 | SEMA6D | 1 |
| PTH | 1 | CD79A | 1 |
| HMG20A | 1 | PTPRZ1 | 1 |
| SLC6A2 | 1 | OR51A4 | 1 |
| FNIP2 | 1 | BACH2 | 1 |
| DACT1 | 1 | CAB39L | 1 |
| TRPV1 | 1 | ST7L | 1 |
| SYNRG | 1 | CBFB | 1 |
| FAM83B | 1 | ATP8A2 | 1 |
| KIAA1549 | 1 | LTBP1 | 1 |
| MOCS1 | 1 | GAK | 1 |
| CHIC1 | 1 | ROBO2 | 1 |
| ACOX1 | 1 | KCNK12 | 1 |
| GSDME | 1 | CNTNAP1 | 1 |
| DCST1 | 1 | WDR24 | 1 |
| HCN1 | 1 | PCDH17 | 1 |
| PDIA4 | 1 | VCAN | 1 |
| FKBP5 | 1 | PSMB2 | 1 |
| TAB1 | 1 | NRXN2 | 1 |
| SHMT1 | 1 | GPR34 | 1 |
| CD2 | 1 | USP6 | 1 |
| DEFB110 | 1 | ETS2 | 1 |
| MAP4K4 | 1 | FAM161B | 1 |
| PPARG | 1 | UBE2D3 | 1 |
| IDH3A | 1 | MYD88 | 1 |
| USP40 | 1 | H3C2 | 1 |
| PPP1R16A | 1 | PAMR1 | 1 |
| MARCO | 1 | PRDM8 | 1 |
| MEX3A | 1 | FAT3 | 1 |
| ARHGAP11A | 1 | ITPR1 | 1 |
| LINGO1 | 1 | LRRFIP2 | 1 |
| BIRC2 | 1 | ADGRA3 | 1 |
| DSPP | 1 | POLG | 1 |
| APPL1 | 1 | AMBRA1 | 1 |
| PADI1 | 1 | MTHFR | 1 |
| AFF1 | 1 | GDI1 | 1 |
| KLF4 | 1 | TPP2 | 1 |
| DNAJC7 | 1 | THOC7 | 1 |
| STK3 | 1 | MCL1 | 1 |
| SERPINC1 | 1 | DEFA3 | 1 |
| Gene | Number of mutations | Gene | Number of mutations |
| EFHC1 | 1 | HSPB2 | 1 |
| NEBL | 1 | EDA | 1 |
| WDR38 | 1 | TRIM11 | 1 |
| PSEN1 | 1 | ITPRID2 | 1 |
| PRSS53 | 1 | TCN1 | 1 |
| TNKS2 | 1 | BRD4 | 1 |
| PODN | 1 | NHS | 1 |
| PEX19 | 1 | FBLN1 | 1 |
| CADM2 | 1 | SERPINB2 | 1 |
| TRAF7 | 1 | NEK10 | 1 |
| APOB | 1 | TLR3 | 1 |
| ZNF512B | 1 | H2BC21 | 1 |
| SPHKAP | 1 | GAS6 | 1 |
| SHLD2 | 1 | INPP5B | 1 |
| RIN2 | 1 | FAM47A | 1 |
| C18orf25 | 1 | GGPS1 | 1 |
| HDAC9 | 1 | ZNF607 | 1 |
| GLRB | 1 | SLCO1C1 | 1 |
| ROBO3 | 1 | PKN2 | 1 |
| ZNF544 | 1 | AVPR1B | 1 |
| JPT1 | 1 | RASIP1 | 1 |
| BCL11A | 1 | MYOM2 | 1 |
| STRIP2 | 1 | TNC | 1 |
| ZNF648 | 1 | ARC | 1 |
| LPIN2 | 1 | SIPA1 | 1 |
| VPS13B | 1 | CCDC158 | 1 |
| IGLV1-47 | 1 | BEND5 | 1 |
| PHLPP2 | 1 | IFT172 | 1 |
| SSTR2 | 1 | TAS2R14 | 1 |
| MOB2 | 1 | INTS12 | 1 |
| IFT80 | 1 | TNKS | 1 |
| ELAVL1 | 1 | ZNF300 | 1 |
| TIGD2 | 1 | DAB2 | 1 |
| BIRC3 | 1 | HCN4 | 1 |
| ZC4H2 | 1 | OR4D10 | 1 |
| SIN3A | 1 | ZNF251 | 1 |
| CHST11 | 1 | SLX4 | 1 |
| REEP4 | 1 | FBF1 | 1 |
| SULT1C2 | 1 | CST3 | 1 |
| FBN2 | 1 | CSDE1 | 1 |
| FAM117A | 1 | SLC24A1 | 1 |
| DACH2 | 1 | JCHAIN | 1 |
| Gene | Number of mutations | Gene | Number of mutations |
| ENTPD5 | 1 | PRRT4 | 1 |
| ORC5 | 1 | RIMBP2 | 1 |
| SLC12A9 | 1 | ZNF292 | 1 |
| EGFR | 1 | NFIX | 1 |
| LIMD1 | 1 | ENAM | 1 |
| CNGB1 | 1 | RNF212 | 1 |
| AGA | 1 | DMBT1 | 1 |
| TEX15 | 1 | NGEF | 1 |
| TECPR1 | 1 | TAP1 | 1 |
| TSC22D1 | 1 | IL22RA2 | 1 |
| AGAP1 | 1 | GASK1B | 1 |
| BLM | 1 | TNS1 | 1 |
| SPTLC3 | 1 | PRDM9 | 1 |
| CHST5 | 1 | THOC5 | 1 |
| NAA25 | 1 | OR2T34 | 1 |
| FAF1 | 1 | LRRN1 | 1 |
| IRF5 | 1 | ESPN | 1 |
| ASIC5 | 1 | BAIAP2L1 | 1 |
| E2F1 | 1 | SLC28A3 | 1 |
| SLC16A3 | 1 | MLXIP | 1 |
| XBP1 | 1 | ASPM | 1 |
| QARS1 | 1 | NBPF3 | 1 |
| TRIM25 | 1 | C12orf66 | 1 |
| P4HA3 | 1 | OPRM1 | 1 |
| STRAP | 1 | HPDL | 1 |
| DNAAF4 | 1 | ISLR2 | 1 |
| NRXN3 | 1 | RHOBTB1 | 1 |
| MYH8 | 1 | H1-5 | 1 |
| CCT6A | 1 | SCAMP5 | 1 |
| CD320 | 1 | KIAA0319 | 1 |
| AFDN | 1 | ZSWIM4 | 1 |
| HSF2BP | 1 | LRRC30 | 1 |
| ARAP3 | 1 | CTSL | 1 |
| ZNF436 | 1 | EIF3E | 1 |
| CCDC47 | 1 | AC008878.1 | 1 |
| PREX2 | 1 | GRM2 | 1 |
| PEAR1 | 1 | TTC39C | 1 |
| LEPR | 1 | PCDHA10 | 1 |
| UBE2R2 | 1 | MRPL19 | 1 |
| RC3H1 | 1 | AC004593.2 | 1 |
| FLRT1 | 1 | VPS9D1 | 1 |
| STIM2 | 1 | AHCYL2 | 1 |
| Gene | Number of mutations | Gene | Number of mutations |
| SLC30A4 | 1 | PRDM2 | 1 |
| PLPPR4 | 1 | IGHV4-31 | 1 |
| CLEC1A | 1 | ZNF695 | 1 |
| PCDHA13 | 1 | SLAMF6 | 1 |
| OR2A7 | 1 | MYBL2 | 1 |
| MYL6B | 1 | ABLIM2 | 1 |
| KCNK3 | 1 | SENP2 | 1 |
| HOXD1 | 1 | ATP10D | 1 |
| MORC4 | 1 | SLCO4C1 | 1 |
| SDHAF2 | 1 | OR13C5 | 1 |
| EIF3A | 1 | MLLT6 | 1 |
| KCNT2 | 1 | PRAMEF4 | 1 |
| DCAF6 | 1 | CEP104 | 1 |
| TBL1XR1 | 1 | ITGB4 | 1 |
| PTPN6 | 1 | COG4 | 1 |
| DNTTIP2 | 1 | TRAM1L1 | 1 |
| ADGRL2 | 1 | LRTM2 | 1 |
| CHKA | 1 | TBRG1 | 1 |
| WDR70 | 1 | RASA3 | 1 |
| TRADD | 1 | SLC1A7 | 1 |
| NKAPL | 1 | GRIA2 | 1 |
| NBAS | 1 | PTPN12 | 1 |
| MUC15 | 1 | RASSF10 | 1 |
| ZNF286A | 1 | F7 | 1 |
| SLIT2 | 1 | HK2 | 1 |
| ZNF568 | 1 | BAAT | 1 |
| DIP2C | 1 | LMTK3 | 1 |
| RAI1 | 1 | GPCPD1 | 1 |
| BAZ2B | 1 | SEMA3D | 1 |
| ZNF285 | 1 | RNF128 | 1 |
| DNAJC9 | 1 | LINGO3 | 1 |
| NEUROD6 | 1 | GGA3 | 1 |
| METTL16 | 1 | PITRM1 | 1 |
| INPP4B | 1 | MARCHF7 | 1 |
| CYLD | 1 | PAX4 | 1 |
| RPTOR | 1 | NAP1L5 | 1 |
| GTDC1 | 1 | DHRS3 | 1 |
| CARD9 | 1 | PRR12 | 1 |
| ZNF658 | 1 | SHANK3 | 1 |
| NACC2 | 1 | SYNPO2 | 1 |
| CBLB | 1 | CLCA2 | 1 |
| LDOC1 | 1 | PTPRR | 1 |
| Gene | Number of mutations | Gene | Number of mutations |
| ZNF345 | 1 | ZNF503 | 1 |
| GLIS3 | 1 | LCMT2 | 1 |
| STXBP5 | 1 | SYNE2 | 1 |
| MN1 | 1 | AFF2 | 1 |
| BMP2 | 1 | COMP | 1 |
| OR6C74 | 1 | CX3CL1 | 1 |
| COL8A1 | 1 | MST1R | 1 |
| CACTIN | 1 | UHRF1 | 1 |
| H4C8 | 1 | SEMA4B | 1 |
| IGKV1-12 | 1 | PPP2R5A | 1 |
| NTRK3 | 1 | PEX5L | 1 |
| CDH2 | 1 | MYOZ1 | 1 |
| PARP12 | 1 | PSMG2 | 1 |
| TDRD6 | 1 | CD300LB | 1 |
| LGALS8 | 1 | H4C4 | 1 |
| PANK4 | 1 | DIRAS1 | 1 |
| TBATA | 1 | OR51A2 | 1 |
| DPYS | 1 | FBXO15 | 1 |
| TRIM41 | 1 | ZNF560 | 1 |
| PDP1 | 1 | SOGA1 | 1 |
| SYTL5 | 1 | CNOT3 | 1 |
| SLC17A4 | 1 | H4C12 | 1 |
| TUB | 1 | ZNF836 | 1 |
| CA3 | 1 | ZNF598 | 1 |
| HS3ST5 | 1 | CHD1L | 1 |
| APBB1 | 1 | OR5AK2 | 1 |
| MAEA | 1 | SYNJ2 | 1 |
| CDKL2 | 1 | PIP5K1A | 1 |
| TGS1 | 1 | SOX18 | 1 |
| SMAD9 | 1 | GABRQ | 1 |
| EDNRA | 1 | AP5Z1 | 1 |
| SPATA17 | 1 | MAP1A | 1 |
| TET1 | 1 | CFAP47 | 1 |
| VPS51 | 1 | CSNK1G3 | 1 |
| GTF3C1 | 1 | IGSF3 | 1 |
| ALX1 | 1 | USP4 | 1 |
| PLCXD3 | 1 | MAF | 1 |
| NFATC2 | 1 | GLB1L3 | 1 |
| GGT1 | 1 | SOWAHD | 1 |
| KCNA6 | 1 | IL6ST | 1 |
| GP9 | 1 | IL2RA | 1 |
| POM121L12 | 1 | OR8J1 | 1 |
| Gene | Number of mutations | Gene | Number of mutations |
| MUC17 | 1 | TBXA2R | 1 |
| DCSTAMP | 1 | SAMD8 | 1 |
| OR4C13 | 1 | SPATA5 | 1 |
| RBM5 | 1 | AL645941.2 | 1 |
| COPS3 | 1 | HCN2 | 1 |
| EXPH5 | 1 | CDH18 | 1 |
| MRPS27 | 1 | TBC1D10C | 1 |
| RNF10 | 1 | FAM32A | 1 |
| RBBP8NL | 1 | PTPRN2 | 1 |
| CSPP1 | 1 | FAM50A | 1 |
| TRPM5 | 1 | KRT79 | 1 |
| GPR149 | 1 | ACACB | 1 |
| EPHA3 | 1 | FREM1 | 1 |
| ZNF439 | 1 | SLC35A2 | 1 |
| TAF1B | 1 | ZNF492 | 1 |
| SCGN | 1 | TMEM132A | 1 |
| WSCD1 | 1 | IGKV2D-30 | 1 |
| GPC5 | 1 | FILIP1 | 1 |
| ATRNL1 | 1 | KCNQ3 | 1 |
| PDE10A | 1 | C2orf78 | 1 |
| CRLF3 | 1 | PTGS1 | 1 |
| ANK2 | 1 | STAT1 | 1 |
| ATG4A | 1 | KMT2C | 1 |
| OR5M8 | 1 | ZNF555 | 1 |
| UCKL1 | 1 | CLCN3 | 1 |
| PRELID3A | 1 | DYNC2H1 | 1 |
| ARID4B | 1 | CPS1 | 1 |
| TFAP2D | 1 | TTLL10 | 1 |
| RSBN1L | 1 | TAGLN3 | 1 |
| GABRA3 | 1 | PICALM | 1 |
| DHRS2 | 1 | RUNX3 | 1 |
| CAND2 | 1 | DNAH2 | 1 |
| SNRPN | 1 | NLRP5 | 1 |
| OR8G5 | 1 | CASC3 | 1 |
| TNFRSF8 | 1 | ABCC10 | 1 |
| AIFM2 | 1 | VIPAS39 | 1 |
| ATPAF1 | 1 | ABCF1 | 1 |
| DLC1 | 1 | CCDC178 | 1 |
| CCDC146 | 1 | CCDC60 | 1 |
| ASH2L | 1 | KCNN4 | 1 |
| THRA | 1 | GOLGA4 | 1 |
| SYCP1 | 1 | TRIM23 | 1 |
| Gene | Number of mutations | Gene | Number of mutations |
| MAGEC3 | 1 | SLC25A47 | 1 |
| TNS2 | 1 | CCDC71 | 1 |
| TTC21A | 1 | LYST | 1 |
| PRDM16 | 1 | ARHGAP45 | 1 |
| SYNPR | 1 | PIK3CD | 1 |
| PCDHGB4 | 1 | IKZF2 | 1 |
| DIPK1C | 1 | TEX10 | 1 |
| SDCBP2 | 1 | CEP63 | 1 |
| IL6 | 1 | MAPKAPK2 | 1 |
| RBM38 | 1 | UAP1L1 | 1 |
| NUP98 | 1 | ARHGAP9 | 1 |
| AMT | 1 | CCR8 | 1 |
| KCTD19 | 1 | H4C11 | 1 |
| SCRIB | 1 | PDLIM4 | 1 |
| CEP70 | 1 | MYO1F | 1 |
| MCM10 | 1 | SETD1A | 1 |
| SPAG16 | 1 | SH3BP4 | 1 |
| METTL1 | 1 | ZNF233 | 1 |
| STPG4 | 1 | IL37 | 1 |
| BCL2A1 | 1 | CBFA2T3 | 1 |
| VPS8 | 1 | PRCC | 1 |
| HIF1AN | 1 | LRRC4 | 1 |
| PDPK1 | 1 | MYO18B | 1 |
| DAB1 | 1 | FASN | 1 |
| PLXNB3 | 1 | ANO4 | 1 |
| KCNU1 | 1 | ADAMTS9 | 1 |
| SLC22A9 | 1 | HELZ | 1 |
| CCDC170 | 1 | EIF1 | 1 |
| IGHV1-24 | 1 | TSPAN31 | 1 |
| NAA11 | 1 | OR2T12 | 1 |
| EIF4A2 | 1 | TBC1D10B | 1 |
| CYP4V2 | 1 | FZD10 | 1 |
| DOCK7 | 1 | DNAH1 | 1 |
| ZNF425 | 1 | FOXP4 | 1 |
| KCNH5 | 1 | ASXL1 | 1 |
| AMER1 | 1 | LGALS3BP | 1 |
| C1S | 1 | TBX18 | 1 |
| ANO7 | 1 | NCBP2L | 1 |
| SETD1B | 1 | MAP1B | 1 |
| ZNF721 | 1 | LUC7L2 | 1 |
| SLC2A5 | 1 | MEGF8 | 1 |
| PXDN | 1 | TTC38 | 1 |
| Gene | Number of mutations | Gene | Number of mutations |
| DOC2A | 1 | DYNC1H1 | 1 |
| FBN1 | 1 | AC242842.3 | 1 |
| CLASP1 | 1 | MSANTD3 | 1 |
| RTEL1 | 1 | DNAH11 | 1 |
| ZIM3 | 1 | PTPN4 | 1 |
| SAFB2 | 1 | GAL3ST1 | 1 |
| CDC5L | 1 | PPP1R36 | 1 |
| VCPKMT | 1 | PTHLH | 1 |
| NAA15 | 1 | FSD1 | 1 |
| CBX6 | 1 | FZD2 | 1 |
| FGFR2 | 1 | NEK11 | 1 |
| ABCA8 | 1 | SRCAP | 1 |
| TENT4A | 1 | ARPP21 | 1 |
| NOTCH1 | 1 | EBLN2 | 1 |
| HTR1B | 1 | AKAP4 | 1 |
| TNPO1 | 1 | A2M | 1 |
| NFYA | 1 | H3C7 | 1 |
| OR13C9 | 1 | OR4S1 | 1 |
| HS6ST3 | 1 | TRIM46 | 1 |
| ITCH | 1 | EIF2B4 | 1 |
| CAMKMT | 1 | PCDH1 | 1 |
| HLA-DMB | 1 | PTGS2 | 1 |
| PPP4C | 1 | PXDNL | 1 |
| COL1A1 | 1 | UGT2B17 | 1 |
| ITGA3 | 1 | RBM42 | 1 |
| BSN | 1 | ZNF479 | 1 |
| SNED1 | 1 | HNRNPD | 1 |
| FUT4 | 1 | ASAP1 | 1 |
| KALRN | 1 | MCHR1 | 1 |
| CNOT2 | 1 | UNC5B | 1 |
| H3C4 | 1 | ANXA11 | 1 |
| NR0B1 | 1 | BLOC1S6 | 1 |
| PHF20 | 1 | DDX47 | 1 |
| OR4D5 | 1 | MEGF6 | 1 |
| PSG8 | 1 | THNSL1 | 1 |
| ARHGEF1 | 1 | YY1AP1 | 1 |
| CARD17 | 1 | CYP3A43 | 1 |
| ZNF521 | 1 | CLMN | 1 |
| PRF1 | 1 | CYP4F3 | 1 |
| FRMPD4 | 1 | CDKL1 | 1 |
| PKD1L1 | 1 | TAS2R30 | 1 |
| SLC4A7 | 1 | CACNA2D2 | 1 |
| Gene | Number of mutations | Gene | Number of mutations |
| SERPINH1 | 1 | TLX1 | 1 |
| TPO | 1 | SPATA31D1 | 1 |
| DPP10 | 1 | FAM102A | 1 |
| MYH7B | 1 | JAKMIP3 | 1 |
| PCCB | 1 | RB1 | 1 |
| AKAP17A | 1 | TRMT5 | 1 |
| SCRN2 | 1 | ERCC2 | 1 |
| RFC4 | 1 | TAS2R1 | 1 |
| OR4D1 | 1 | FRMD4B | 1 |
| FIGN | 1 | NUP188 | 1 |
| IGSF9B | 1 | ZNF281 | 1 |
| MICAL2 | 1 | DGLUCY | 1 |
| NFKB2 | 1 | PNPLA5 | 1 |
| PLA2G5 | 1 | PAPPA | 1 |
| IER3 | 1 | OBSCN | 1 |
| MYBPC2 | 1 | BAHCC1 | 1 |
| NKX6-3 | 1 | PRSS16 | 1 |
| MIA2 | 1 | VCPIP1 | 1 |
| CYP24A1 | 1 | GNAI2 | 1 |
| HOXC13 | 1 | OCRL | 1 |
| MAMLD1 | 1 | SHANK2 | 1 |
| OTOP2 | 1 | FNDC4 | 1 |
| PLA2G2E | 1 | MYF6 | 1 |
| CXCR5 | 1 | NPR2 | 1 |
| IFIH1 | 1 | PIAS1 | 1 |
| IGHD6-13 | 1 | HOXC9 | 1 |
| UNC5D | 1 | EXTL3 | 1 |
| TTK | 1 | GNB1L | 1 |
| TRNT1 | 1 | OR52N5 | 1 |
| C11orf86 | 1 | PHLDB1 | 1 |
| OR14I1 | 1 | IGKV3D-11 | 1 |
| RXFP2 | 1 | PHPT1 | 1 |
| OSTM1 | 1 | GBP1 | 1 |
| ZNF160 | 1 | RFX2 | 1 |
| DUSP13 | 1 | RPS13 | 1 |
| TESC | 1 | NCAN | 1 |
| NT5DC3 | 1 | SLA | 1 |
| DAGLA | 1 | AC127029.3 | 1 |
| DTX1 | 1 | GTPBP6 | 1 |
| MAP1S | 1 | PASK | 1 |
| POU2F2 | 1 | H2BC18 | 1 |
| ZNF644 | 1 | KIF3B | 1 |
| Gene | Number of mutations | Gene | Number of mutations |
| ABCA4 | 1 | C17orf97 | 1 |
| PLD1 | 1 | TAGAP | 1 |
| CPSF1 | 1 | ANKRD30A | 1 |
| OTOP1 | 1 | RPH3A | 1 |
| DCLK3 | 1 | USP24 | 1 |
| PTPRK | 1 | H2AZ2 | 1 |
| ABHD17B | 1 | H2AC20 | 1 |
| EP300 | 1 | PAM | 1 |
| ABT1 | 1 | BMP3 | 1 |
| TAS2R16 | 1 | EZH2 | 1 |
| GRIN2D | 1 | COL11A1 | 1 |
| OPN1SW | 1 | KLHDC4 | 1 |
| WNK3 | 1 | STAU1 | 1 |
| MYT1L | 1 | KRT37 | 1 |
| ATP5MC1 | 1 | CRYBG1 | 1 |
| CHST6 | 1 | ATXN2 | 1 |
| RYR2 | 1 | JAG2 | 1 |
| OR5H2 | 1 | PKMYT1 | 1 |
| CRYGN | 1 | TMEM237 | 1 |
| TNRC6A | 1 | PTPN13 | 1 |
| TAOK1 | 1 | AKT2 | 1 |
| SERPINB3 | 1 | EFHB | 1 |
| CCDC185 | 1 | RGS7 | 1 |
| PGGT1B | 1 | ZNF180 | 1 |
| POTEE | 1 | CAVIN1 | 1 |
| IL4I1 | 1 | ABHD12B | 1 |
| TTC21B | 1 | CTNS | 1 |
| SORL1 | 1 | ARAP1 | 1 |
| SLC16A12 | 1 | GPR50 | 1 |
| TXNDC2 | 1 | SLC8A3 | 1 |
| TOP3A | 1 | HDAC7 | 1 |
| LY75 | 1 | GPC1 | 1 |
| COL24A1 | 1 | CHD4 | 1 |
| TJP2 | 1 | DLX6 | 1 |
| SPOCD1 | 1 | CDH9 | 1 |
| ATAD3A | 1 | UBE3B | 1 |
| EXOSC5 | 1 | DLG2 | 1 |
| ZNF677 | 1 | ZFHX3 | 1 |
| HELZ2 | 1 | PIK3C2G | 1 |
| PYY | 1 | SEMA6B | 1 |
| POLD1 | 1 | H3-3A | 1 |
| SHISAL1 | 1 | GPRASP1 | 1 |
| Gene | Number of mutations | Gene | Number of mutations |
| CCDC130 | 1 | ZNF474 | 1 |
| OR5D16 | 1 | SLC27A6 | 1 |
| CDC25B | 1 | PCYOX1L | 1 |
| ROS1 | 1 | UVSSA | 1 |
| KRTAP11-1 | 1 | ANKS1A | 1 |
| PSKH2 | 1 | CLSTN2 | 1 |
| MED24 | 1 | ZC3HC1 | 1 |
| TLR4 | 1 | TNKS1BP1 | 1 |
| IGHV3-35 | 1 | GPR84 | 1 |
| ERICH3 | 1 | SYNGR4 | 1 |
| TTLL6 | 1 | ZPLD1 | 1 |
| ZFP36L1 | 1 | PRPF19 | 1 |
| SMARCA5 | 1 | KRT23 | 1 |
| STAT3 | 1 | FBXO24 | 1 |
| AURKB | 1 | PRDM1 | 1 |
| CLCN1 | 1 | GIGYF2 | 1 |
| ZNF142 | 1 | CERS6 | 1 |
| METTL25 | 1 | GON4L | 1 |
| MMP15 | 1 | AMOTL2 | 1 |
| PIM2 | 1 | SPANXN2 | 1 |
| PRAMEF10 | 1 | HS3ST1 | 1 |
| DOCK3 | 1 | SPRY3 | 1 |
| KY | 1 | CHD1 | 1 |
| SLC19A1 | 1 | U2SURP | 1 |
| OR2M2 | 1 | MICAL3 | 1 |
| VWF | 1 | NCKAP1 | 1 |
| FAS | 1 | TADA2B | 1 |
| ABLIM3 | 1 | XKR4 | 1 |
| IGLV3-10 | 1 | OR10H3 | 1 |
| OR4D2 | 1 | ZBED1 | 1 |
| SLITRK3 | 1 | SHROOM2 | 1 |
| SAMHD1 | 1 | MYBL1 | 1 |
| PTPRU | 1 | CDH3 | 1 |
| VPS13C | 1 | SYMPK | 1 |
| KMT2A | 1 | HAO1 | 1 |
| PLEKHH2 | 1 | ECSCR | 1 |
| DNAH7 | 1 | NEDD4 | 1 |
| FAM9A | 1 | COASY | 1 |
| CD68 | 1 | GPR25 | 1 |
| TMEM245 | 1 | UNC5C | 1 |
| CHD8 | 1 | HMGB2 | 1 |
| AHR | 1 | RAP1GAP2 | 1 |
| Gene | Number of mutations | Gene | Number of mutations |
| TAF6 | 1 | ACTG1 | 1 |
| G2E3 | 1 | TULP4 | 1 |
| CCDC191 | 1 | ARHGEF2 | 1 |
| CD163L1 | 1 | LRP1 | 1 |
| NKTR | 1 | SORCS3 | 1 |
| TAB3 | 1 | NFYC | 1 |
| ADGRL3 | 1 | FRMPD1 | 1 |
| ZNF649 | 1 | NUTM1 | 1 |
| PAPOLA | 1 | OR5B21 | 1 |
| INHBA | 1 | POLR2J3 | 1 |
| RBPJL | 1 | ENTHD1 | 1 |
| CST4 | 1 | XPO6 | 1 |
| SMC4 | 1 | NCAPG | 1 |
| PCDHA2 | 1 | PLK2 | 1 |
| TASOR | 1 | SSTR4 | 1 |
| SIX4 | 1 | RPS6KA6 | 1 |
| AATF | 1 | PTN | 1 |
| FSCB | 1 | HS6ST2 | 1 |
| EXOC7 | 1 | EIF2B5 | 1 |
| DENND3 | 1 | NR2E3 | 1 |
| CELSR1 | 1 | ELOVL6 | 1 |
| IGHV3-30 | 1 | NBR1 | 1 |
| LAMA3 | 1 | CYTH3 | 1 |
| CUBN | 1 | SRSF1 | 1 |
| COL4A5 | 1 | NDUFS1 | 1 |
| LACTB | 1 | DPYD | 1 |
| ARIH2 | 1 | IRX2 | 1 |
| OR5R1 | 1 | SCAP | 1 |
| PSPH | 1 | ACOT4 | 1 |
| SRM | 1 | TMEM131L | 1 |
| KLHL2 | 1 | SERPIND1 | 1 |
| TNFSF13B | 1 | MIB2 | 1 |
| FAM13C | 1 | RYK | 1 |
| MTUS1 | 1 | HELB | 1 |
| RHOBTB2 | 1 | CYP11B1 | 1 |
| TMEM132D | 1 | KCNT1 | 1 |
| PCED1A | 1 | RHOT1 | 1 |
| H2BC13 | 1 | PPP1R27 | 1 |
| FPGT | 1 | SLC35F2 | 1 |
| H2BC10 | 1 | GLP2R | 1 |
| ANO1 | 1 | FANCM | 1 |
| CAPN7 | 1 | ETFRF1 | 1 |
| Gene | Number of mutations | Gene | Number of mutations |
| GOPC | 1 | PCDHB10 | 1 |
| PLG | 1 | HEMK1 | 1 |
| PRRC2A | 1 | IGKV5-2 | 1 |
| PPWD1 | 1 | LRRC73 | 1 |
| RHBDF2 | 1 | C4A | 1 |
| PSG11 | 1 | SLITRK5 | 1 |
| MAOB | 1 | DYSF | 1 |
| UNC13B | 1 | LOX | 1 |
| EXOC3L1 | 1 | CAMSAP2 | 1 |
| EMC1 | 1 | NPY4R | 1 |
| ZNF454 | 1 | PPID | 1 |
| SLC16A1 | 1 | ANK3 | 1 |
| TTYH2 | 1 | MAP2K1 | 1 |
| HCAR2 | 1 | ZNF395 | 1 |
| ABCA10 | 1 | TRPC5 | 1 |
| AKR1B15 | 1 | ZBP1 | 1 |
| IL13RA1 | 1 | NDST3 | 1 |
| ABCA13 | 1 | GLS | 1 |
| OR4C46 | 1 | KIF3C | 1 |
| EPHA7 | 1 | USP39 | 1 |
| CEP290 | 1 | METTL4 | 1 |
| TNK2 | 1 | FAM78B | 1 |
| SNX1 | 1 | POF1B | 1 |
| INTS7 | 1 | RBM12 | 1 |
| TSNARE1 | 1 | CDC42BPG | 1 |
| SLC7A2 | 1 | TECPR2 | 1 |
| OR52A1 | 1 | KIF21A | 1 |
| KRTAP5-3 | 1 | ZCCHC8 | 1 |
| COL17A1 | 1 | HSPA14 | 1 |
| GRIN2C | 1 | NEDD4L | 1 |
| ABI1 | 1 | CXXC1 | 1 |
| PCDHB13 | 1 |  |  |
